# Molecular Striptease of a Thiadiazolopyrimidine Hit Yields a Streamlined, Artifact-Free NADPH Mimic for NOX Inhibition

**DOI:** 10.1021/acsmedchemlett.6c00370

**Published:** 2026-08-08

**Authors:** Emanuele Fabbrizi, Andrea Mancini, Chiara Lambona, Salvatore Macrì, Marianna Nalli, Francesco Quilli, Federico Pandolfi, Domenico Raimondo, Sara Marchese, Andrea Mattevi, Jihyeon Lee, Minhye Choi, Yun Soo Bae, Nadya Hristova-Avakumova, Magdalena Kondeva-Burdina, Denitsa Stefanova, Neda Anastassova, Sergio Valente, Dante Rotili, Antonello Mai

**Affiliations:** † Department of Drug Chemistry and Technologies, 9311 Sapienza University of Rome, P.le Aldo Moro 5, 00185 Rome, Italy; ‡ Department of Molecular Medicine, Sapienza University of Rome, Laboratory Affiliated to Institut Pasteur - Cenci Bolognetti Foundation, Viale Regina Elena 291, 00161 Rome, Italy; § Department of Biology and Biotechnology “Lazzaro Spallanzani”, University of Pavia, 27100 Pavia, Italy; ∥ Department of Life Science, 26717 Ewha Womans University, Seoul 120-750, South Korea; ⊥ Celros Biotech, Rm 405, Science Building C, 52 Ewhayeodae-Gil, Seodaemoon-Gu, Seoul 120-750, South Korea; # Department of Medical Physics and Biophysics, Faculty of Medicine, Medical University of Sofia, 2 Zdrave Str., 1431 Sofia, Bulgaria; ⊗ Laboratory of Drug Metabolism and Drug Toxicity, Department of Pharmacology, Pharmacotherapy and Toxicology, Faculty of Pharmacy, 58789 Medical University-Sofia, 2 Dunav Str., 1000 Sofia, Bulgaria; ○ Institute of Organic Chemistry with Centre of Phytochemistry, 440880 Bulgarian Academy of Sciences, Acad. G. Bonchev Str., Building 9, 1113 Sofia, Bulgaria; ● Department of Science, Roma Tre University, 00146 Rome, Italy; ◇ Biostructures and Biosystems National Institute (INBB), 00165 Rome, Italy

**Keywords:** NOX inhibitors, Thiadiazolopyrimidine, Scaffold truncation, Neuroprotection, Redox liabilities

## Abstract

The convergence of oxidative stress and inflammation drives neurodegeneration and cancer, positioning NADPH oxidases (NOXs) as critical therapeutic targets. Starting from the polyfunctional thiadiazolopyrimidine hit **1**, we systematically truncated its peripheral arms to map minimum pharmacophoric requirements. While extensive clipping compromised activity, strategic optimization yielded the streamlined analogue **5**. In silico, **5** acted as a competitive NADPH mimic; in cell-free assays, it maintained multi-isoform potency, inhibiting NOX1 and NOX5 at low-micromolar concentrations. In rat brain subcellular fractions, **1** and **5** demonstrated concentration-dependent neuroprotective and antioxidant efficacy (1–10 μM) by suppressing lipid peroxidation and preserving mitochondrial and synaptosomal viability. Notably, chemical profiling proved that **5** successfully stripped away the pro-oxidant liabilities and radical-scavenging artifacts inherent to **1**. In cancer, **1** displayed some antiproliferative activity, mainly in hematological malignancies. Compound **5**, although aqueous solubility issues limited its cellular antiproliferative performance, represents a specific, artifact-free architectural starting point for future selective NOX inhibitor development.

## Graphical abstract

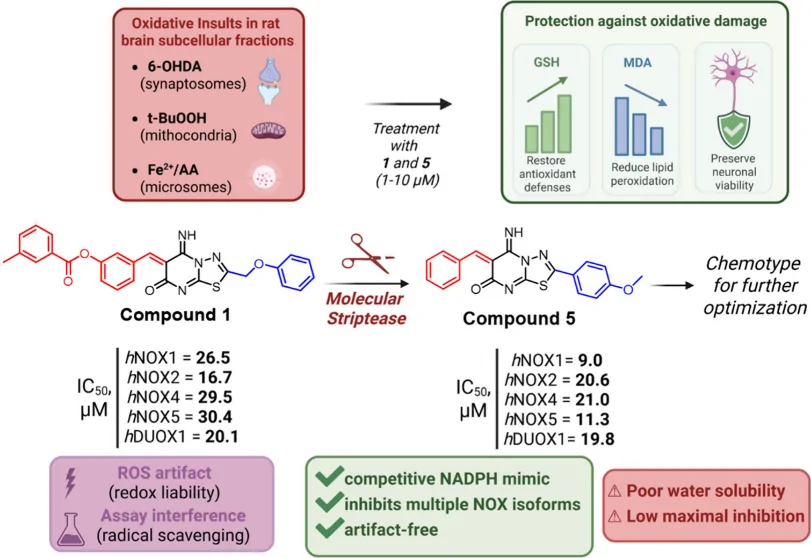

Neurodegenerative disorders and cancer are increasingly recognized as multifactorial pathologies driven by the convergence of oxidative stress and chronic inflammation.^1–4^ Among the enzymatic sources of pathological reactive oxygen species (ROS), NADPH oxidases (NOXs) play a prominent role.^5^ While dysregulated NOX1, NOX2, and NOX4 isoforms are strongly implicated in microglial activation, blood–brain barrier dysfunction, and tumor progression,^6–14^ recent evidence also links calcium-dependent NOX5 and dual oxidase 1 (DUOX1) to neurovascular injury and oncogenic redox-signaling.^15–18^ Despite strong biological validation, developing small-molecule NOX inhibitors suitable for central nervous system (CNS) applications remains challenging.

Recently, via ultralarge in silico screens of about 350 million compounds utilizing VirtualFlow33 against the *Cylindrospermum stagnale* NOX5 (*cs*NOX5) dehydrogenase domain (both FAD-complexed and apo forms),^19^ we identified the synthetic hit **1** (M11, Figure 1).^20^ Compound **1** exhibited (sub)­micromolar inhibition in NOX membrane preparations (IC_50_ values = 0.36–40.2 μM) without structural ROS-scavenging or assay interference.^20^ However, its high structural complexity and suboptimal physicochemical profile prompt further investigation into its minimal pharmacophoric requirements to enable neurotherapeutic optimization.

**Figure 1:**
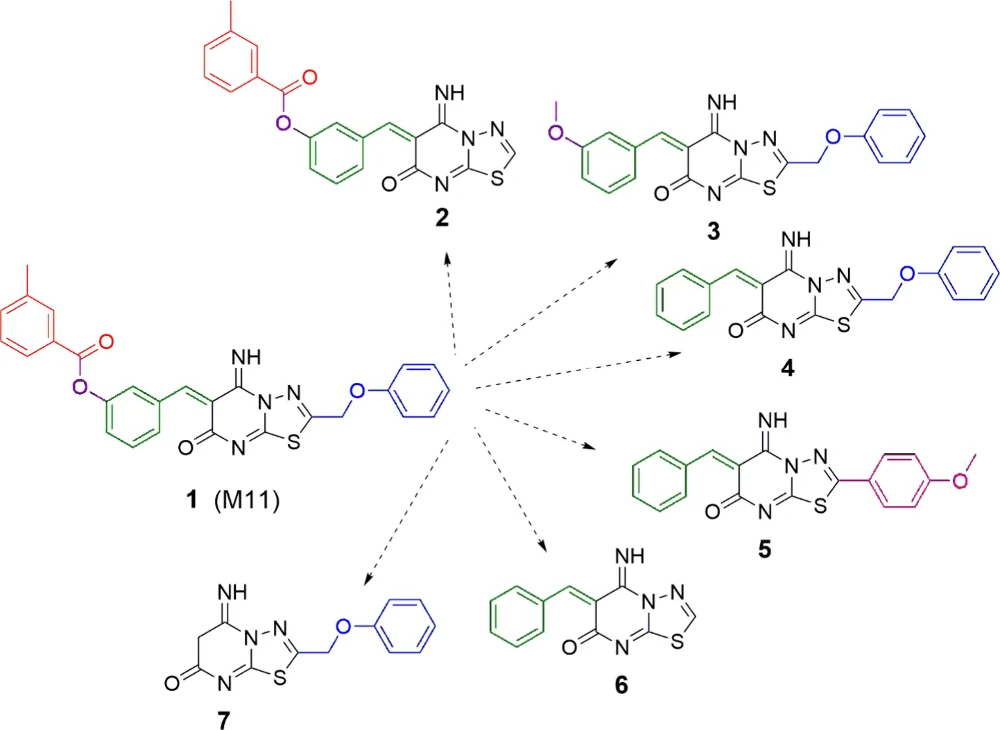
Chemical structures of compound **1** (M11) and simplified analogues **2**–**7**.

To address this, we applied a “molecular striptease” strategy^21,22^ to dissect the contribution of individual structural motifs of **1** to NOX inhibition. We hypothesized that retaining the core NOX-interacting pharmacophore while strategically removing nonessential, bulky elements would preserve inhibitory activity, enhance chemical tractability, and provide clearer structure–activity relationships (SAR) across different NOX family members.

Starting from the multisubstituted core of **1**, we systematically clipped its peripheral arms (Figure 1): deletion of the 2-phenoxymethyl chain from the [1,3,4]­thiadiazolo­[3,2-*a*]­pyrimidine nucleus yielded **2**, while cleavage of the 3-methylbenzoyl moiety from the 3-hydroxybenzylidene substructure afforded **3**. Further truncation of **3** via removal of the 3-benzylidene substituent produced analogues **4** and **5**. Finally, maximizing the structural simplification of **4** by systematically deleting either the phenoxymethyl chain or the benzylidene motif yielded the minimal heterocyclic cores **6** and **7**, respectively.

## Chemistry

The synthesis of **1** (**M11**) and its simplified analogues **2–6** was accomplished starting from the key intermediates 5-imino-5,6-dihydro-7*H*-[1,3,4]­thiadiazolo­[3,2-*a*]­pyrimidin-7-ones (**7–9**). These cores were prepared via cyclocondensation of the corresponding commercially available 1,3,4-thiadiazol-2-amines with ethyl cyanoacetate in anhydrous ethanol containing sodium ethoxide, followed by acidic quenching. Subsequent Knoevenagel condensation of intermediates **7–9** with appropriate aromatic aldehydes in a DMF/EtOH mixture under basic conditions afforded the target compounds **1–6** (Scheme 1). Detailed synthetic protocols and analytical characterization are provided in the Supporting Information.

**Scheme 1:**
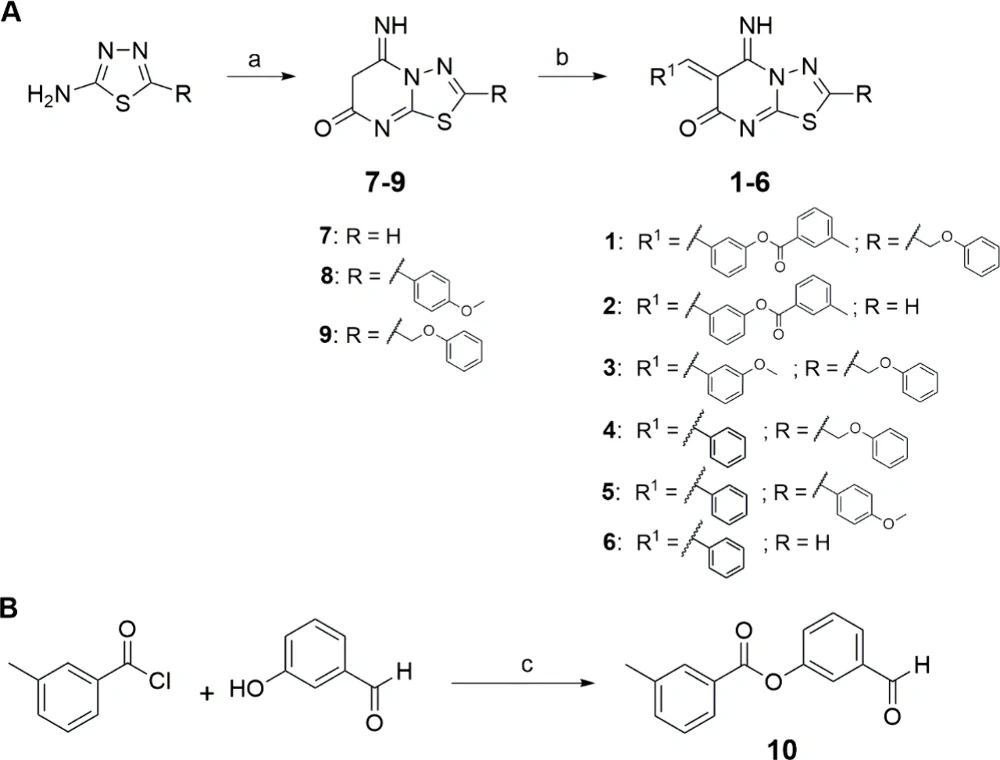
General Synthesis of M11 and its Analogues^a^

a Reagents and conditions: a) Na (1.0 equiv), anhydrous EtOH, ethyl cyanoacetate (1.0 equiv), reflux, 2 h; b) appropriate aromatic aldehyde (1.05 equiv), Et_3_N (cat.), DMF/EtOH (1:1), 50 °C, 3 h; c) Et_3_N, THF, DCM, 0 °C, 1 h.

## Biochemistry and Structure–Activity Relationships (SAR)

Compounds **1–6** and intermediate **7** were evaluated against human NOX isoforms (NOX1, NOX2, NOX4, NOX5, and DUOX1) utilizing purified membrane preparations from transgenic *Drosophila* expressions. ROS production was quantified via lucigenin-amplified chemiluminescence.^23^

In this assay platform, the parental hit **1** displayed IC_50_ values of 26.5 μM (NOX1), 16.7 μM (NOX2), 29.5 μM (NOX4), 30.4 μM (NOX5), and 20.1 μM (DUOX1) (Table 2), confirming its multi-isoform activity profile with minimal 1.5- to 2.5-fold potency variation for NOX1, −2, and −4, and 84-fold lower potency for NOX5, when compared to initial screenings.^20^ Analogues **2–7** were primary-screened at fixed 10 and 100 μM doses (Table 1); IC_50_ values were subsequently extrapolated for derivatives achieving >70% inhibition at 100 μM against at least one isoform (Table 2).

**Table 1:** Percentage of Inhibition of NOX Isoforms at Fixed Doses (10 and 100 μM) by Compounds 1–7

	% inhibition, μM									
	NOX1		NOX2		NOX4		NOX5		DUOX1	
compd	10	100	10	100	10	100	10	100	10	100
**1**	30.7	81.4	41.5	81.9	29.3	82.6	26.3	71.9	31.4	80.8
MC5328										
**2**	7	77.3	14.5	76.6	13.3	76.9	10.2	80.5	12.1	82.9
MC5333										
**3**	20.2	46.0	15.3	51.1	26.4	52.5	14.5	54.7	31.4	56.8
MC5329										
**4**	22.6	64.9	8.1	57.1	3.2	64.2	14.0	65.2	26.1	62.3
MC5330										
**5**	25.6	39.3	18.7	50.8	19.7	50.3	21.9	43.7	25.1	62.9
MC5335										
**6**	6.0	10.3	2.2	5.0	23.9	17.1	7.4	8.0	9.7	6.7
MC5334										
**7**	10.9	4.7	–9.5	1.8	–7.4	3.3	4.4	1.9	5.5	3.4
MC5327										

**Table 2:**
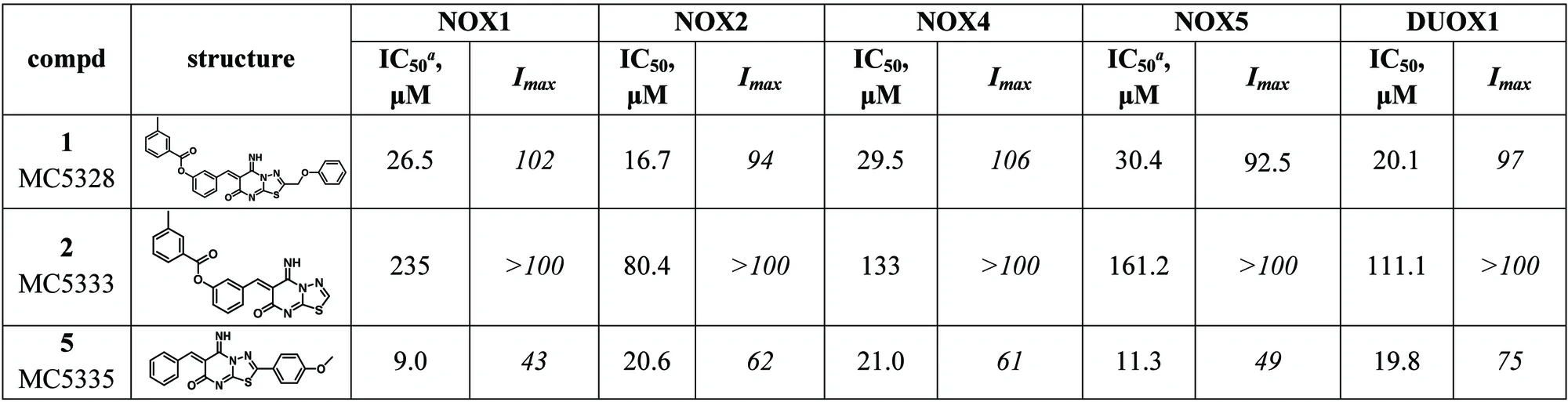
IC_50_ Values and Percentages of Maximum Inhibition (*I*_*max*_) by 1, 2, and 5 against NOX1, NOX2, NOX4, NOX5, and DUOX1

a IC_50,app_ for compound **5**

At fixed concentrations, analogue **2** (lacking the 2-phenoxymethyl chain) retained a profile comparable to **1** against NOX2 and NOX4, whereas the 3-methylbenzoyl-clipped derivative **3** showed reduced efficacy. Among the highly truncated derivatives **4** and **5**, compound **4** exhibited poor activity. Unexpectedly, compound **5** regained prominent inhibitory potency, an effect driven by replacing the phenoxymethyl motif of **4** with a 4-methoxyphenyl group. The minimal heterocyclic cores **6** and **7** were virtually inactive.

Intriguingly, despite high screening inhibition by **2** at 100 μM, its measured IC_50_ values revealed a general drop in potency relative to **1**. Conversely, compound **5** exhibited lower or comparable efficacy to **1** at fixed high doses, bound to limited maximum inhibition (*I*_*max*_) values. This discouraging result likely reflects the poor solubility of **5**, which hampered the compound’s ability to achieve high maximal inhibition values against NOXs. Nevertheless, the apparent IC_50_ (IC_50,app_) values determined for **5** revealed an inhibitory potency comparable to **1** against NOX2, NOX4, and DUOX1, alongside a 2.7- to 2.9-fold increase in potency against NOX1 (IC_50,app_ = 9.0 μM) and NOX5 (IC_50,app_ = 11.3 μM), breaking into the low-micromolar range. The inhibition curves for **1**, **2**, and **5** against the tested NOX isoforms are reported in Figure S1 in Supporting Information.

## Molecular Modeling and Binding Determinants of Compound 5

To elucidate the structural determinants driving the potency of the simplified analogue **5**, we cross-compared its predicted binding modes against NOX1 and NOX5 with those of **1**. Protein–ligand complexes were modeled using Boltz2, yielding high-confidence predictions (confidence scores: 0.85 for **5**-NOX5; 0.89 for **1**-NOX5).

Both compounds occupy the catalytic cofactor-binding pocket within the dehydrogenase (DH) domain of NOX5, spanning the interface of the NADPH- and FAD-binding domains (Figure 2A).^24,25^ In NOX5, the binding pose of **5** overlaps extensively with the natural cofactors NADPH/NADP^+^ from the human NOX5 cryo-EM structure (PDB: 8U87)^26^ and the *cs*NOX5 crystal structure complexed with inhibitor M41 (designated as compound 3 in,^20^ PDB: 8CB0) (Figure 3A). This conserved alignment suggests that the simplified architecture of **5** effectively acts as a competitive NADPH mimic.

**Figure 2:**
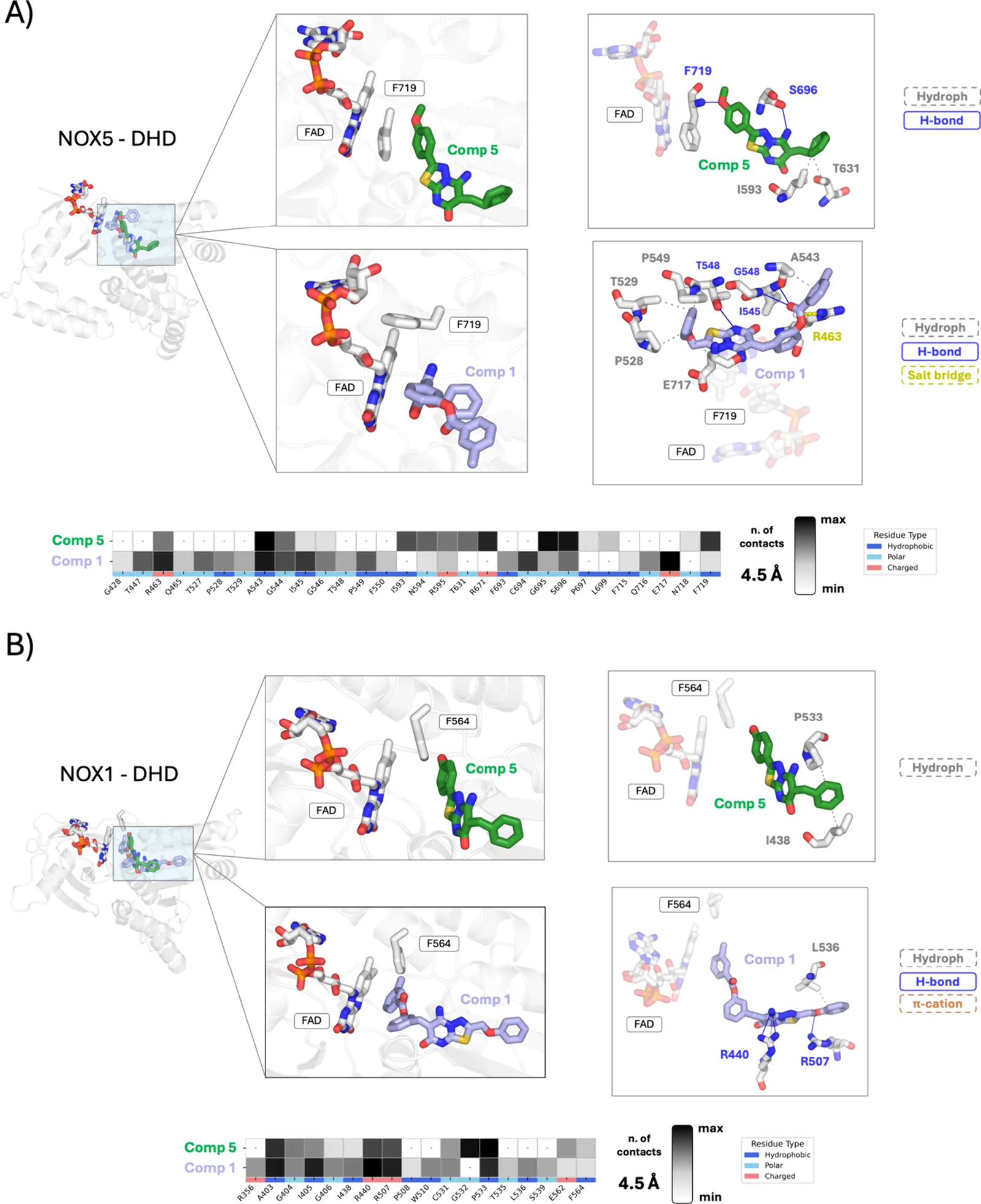
In A) and B), a cartoon representation of the Dehydrogenase domain (DHD) of NOX5 and NOX1 respectively, with zoomed perspectives on the binding mode of compound **5** (in green) and compound **1** (in light blue). The frames on the left represent overall positioning of both compounds relative to C-terminal residue and FAD, while the right frames indicate the computed noncovalent interactions. A comparative protein–ligand contact fingerprint of the compounds is represented below the frames, computed as the number of atoms of each residue that fall within a 4.5 Å cutoff. Missing contacts are represented with a dash.

**Figure 3:**
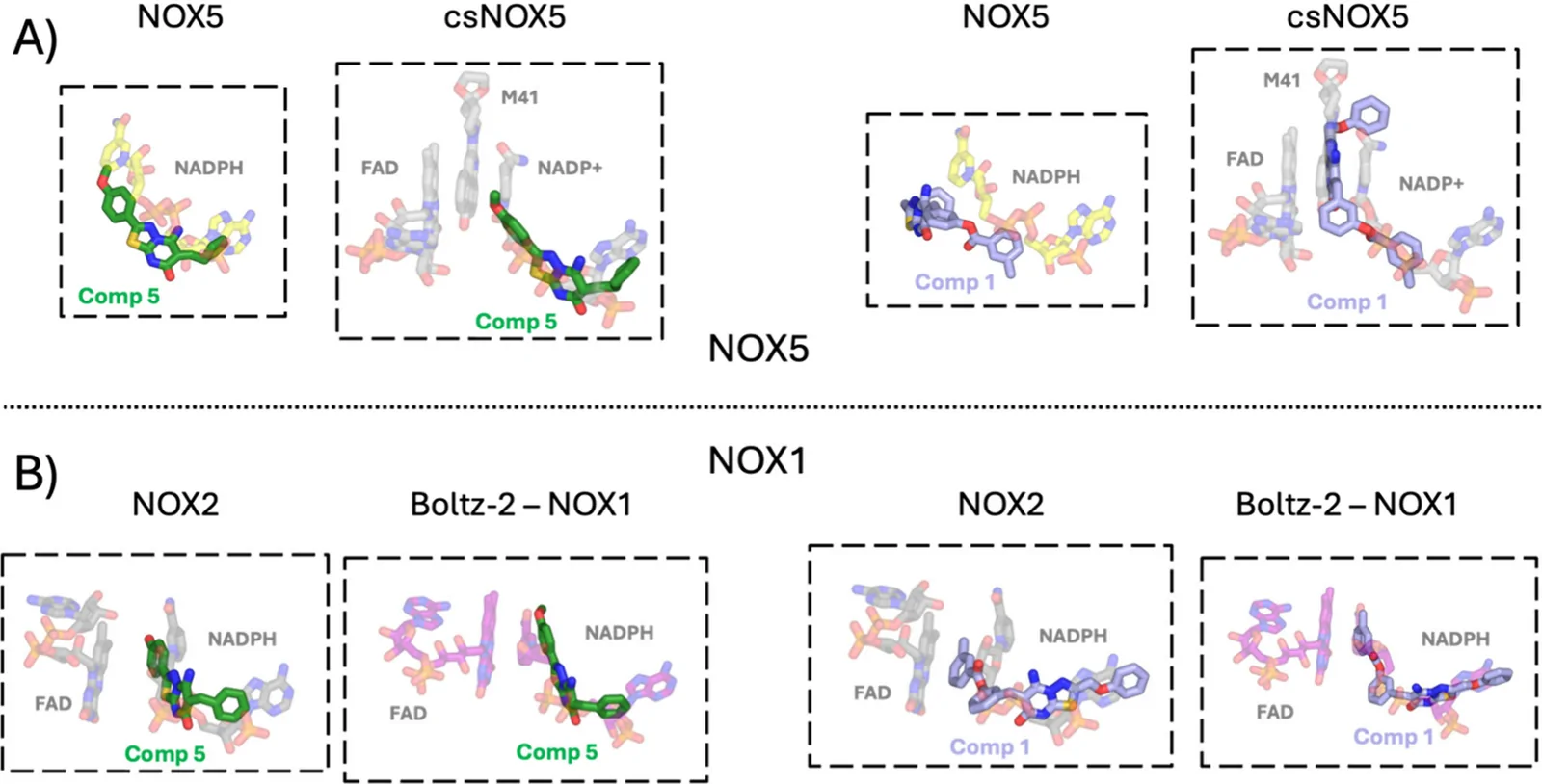
In A) superimpositions of the predicted NOX5-compound **5** and NOX5-compound **1** complexes against the cryo-EM NOX5 (PDB: 8U87) structure and homologous csNOX5-M41 complex (PDB: 8CB0). In B), superimpositions pertaining to the predicted NOX1 complexes, using the cryo-EM structure of NOX2 (PDB: 8WEJ) and a Boltz-2 generated model of the isoform.

Specifically, **5** simultaneously engages the key residues involved in NADPH recognition—R463, R595, and R671—exploiting a crucial, conserved electrostatic network within the DH domain. Additionally, its phenoxymethyl and imino-oxo-pyrimidine motifs form hydrogen bonds with S696 and F719, the latter being a highly conserved C-terminal residue critical for regulating NOX catalytic activity.^27^ Hydrophobic interactions with NBD residues I593 and T631 further stabilize the complex.

Conversely, the bulkier compound **1** inserts deeper toward the FAD cofactor rather than the NADPH site (Figure 3A), partially mimicking M41’s stacking triad and engaging only two of the three key arginines. This indicates a binding mode less focused on NADPH mimicry and more dependent on local structural adaptation.

High-confidence predictions were similarly obtained for NOX1 (scores: 0.89 for **5**-NOX1; 0.88 for **1**-NOX1). In these models (Figure 2B), both ligands occupy the DH domain catalytic region. Compound **5** strictly preserves the NADPH-aligned binding mode observed in NOX5, making no contact with the FAD cofactor within a 4.5 Å cutoff, and is stabilized by hydrophobic interactions with I438 and P533. Compound **1** accommodates a distinct conformation within the pocket, contacting a larger surface area due to its size, forming hydrogen bonds and π-cation interactions with R440 and R507.

In the absence of an experimentally obtained NOX1 structure, superimposition onto the human NOX2 cryo-EM structure (PDB: 8WEJ, ∼60% sequence identity)^28^ confirmed that both ligands align within the solvent-exposed groove normally engaged by NADPH, without penetrating as deeply as **1** does in NOX5 (Figure 3B). To validate this competitive mechanism, a fully cofactored (FAD/NADPH) NOX1 structure was generated via Boltz2 (score: 0.9). The analysis confirmed that **5** substantially overlaps with the core NADPH architecture, while **1** aligns predominantly with its nicotinamide portion via its phenoxymethyl group.

Overall, while **1** exhibits high conformational variability across different NOX isoforms, the simplified analogue **5** maintains a highly consistent, rigid binding mode, supporting its role as a targeted competitive NADPH mimic across the NOX family.

## Neuroprotective, Antioxidant, and Radical Scavenging Profiles

Given the central role of NOXs in neurovascular impairment, compounds **1** and **5** were evaluated for neuroprotective efficacy against oxidative damage in human neuroblastoma SH-SY5Y cells.^29,30^ Stability studies conducted on **1** in PBS at 37 °C after 4, 24, and 48 h showed no significant decrease of compound over the time course, indicating good stability for the benzoic ester of **1** under these conditions (Figure S2 in Supporting Information). MTT assays established favorable cellular tolerance, with IC_50_ values of 57.99 μM (**1**) and 53.02 μM (**5**) (Figure S3 in Supporting Information). Based on these profiles, nontoxic doses (1, 5, and 10 μM) were screened in three complementary models of neuronal injury: H_2_O_2_-induced oxidative stress, l-glutamate-induced excitotoxicity, and MPP^+^-induced neurotoxicity. Neither compound produced a statistically significant protective effect in these cellular setups (data not shown).

To evaluate intrinsic neuroprotective and antioxidant properties beyond cellular models, compounds **1** and **5** were tested on rat brain subcellular fractions (Figures 4–6). In isolated synaptosomes, exposure to 6-hydroxydopamine (6-OHDA) (150 μM) significantly reduced viability and GSH levels by approximately 50%. Co-treatment with **1** or **5** (1–10 μM) concentration-dependently rescued both parameters, with **1** (10 μM) showing the highest efficacy by preserving 78% viability and 40% GSH (Figure 4).

**Figure 4:**
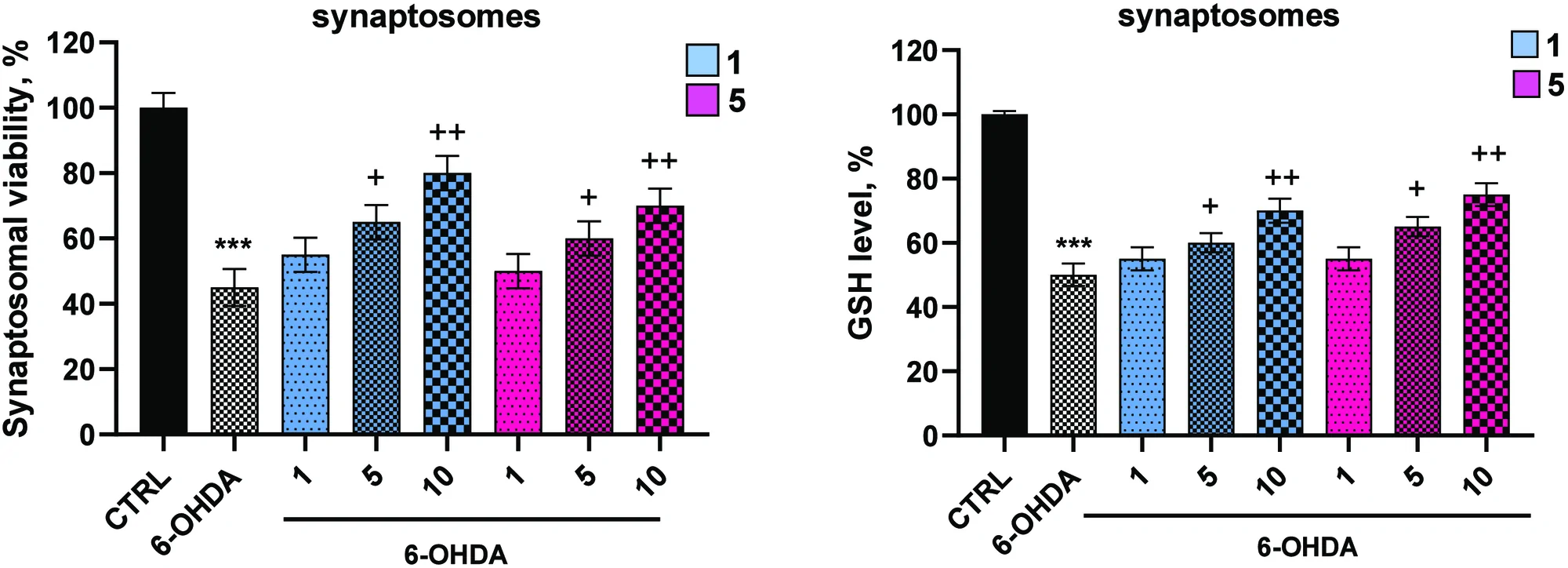
Neuroprotective effects of **1** and **5** (1–10 μM) against 6-OHDA-induced toxicity (150 μM) in rat brain synaptosomes. *P < 0.05; ***P < 0.001 vs control (nontreated synaptosomes). ^+^ *P* < 0.05; ^+2^ *P* < 0.01 vs control (6-OHDA).

In mitochondria, *tert*-butyl hydroperoxide (*t*-BuOOH) (75 μM) depleted GSH by 50% and increased MDA by 150%. Compounds **1** and **5** (1–10 μM) mitigated this oxidative damage; at 10 μM, **1** outperformed **5**, restoring GSH by 40% and suppressing MDA production by 60% (Figure 5).

**Figure 5:**
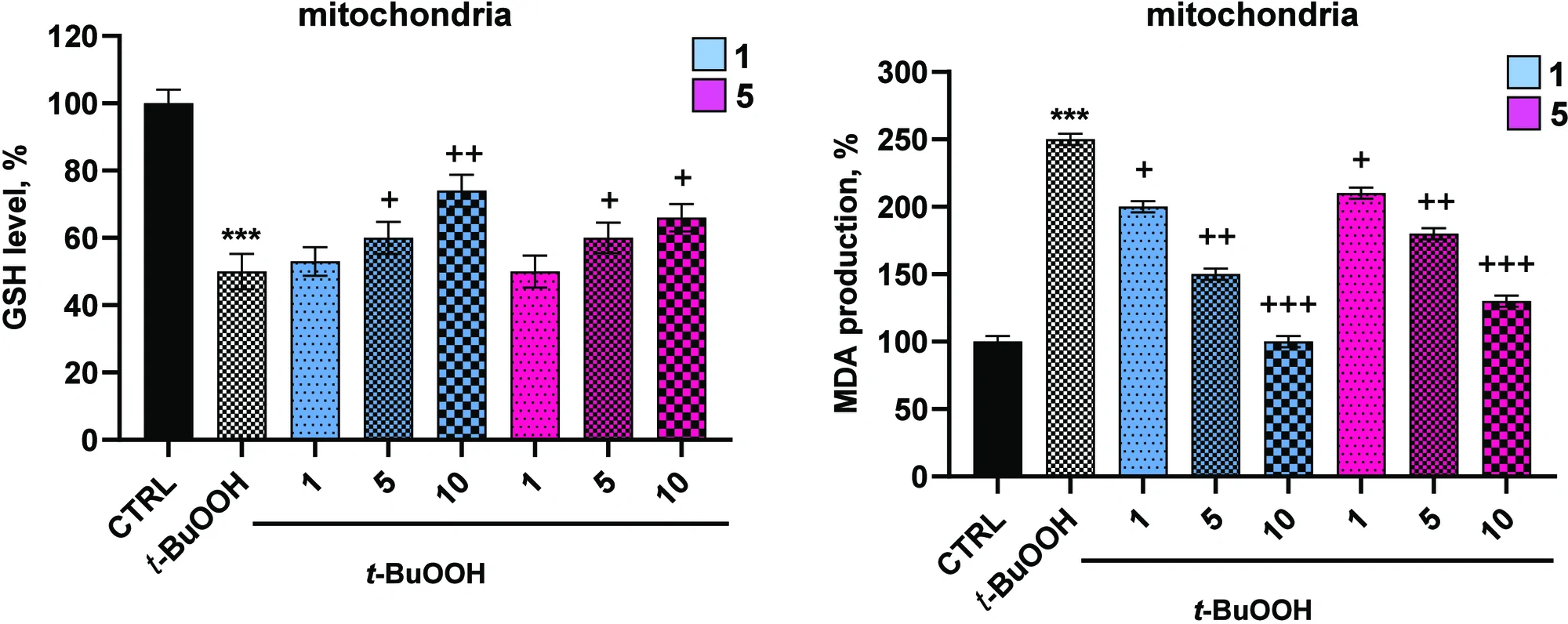
Effects of **1** and **5** (1–10 μM) on GSH and MDA levels in *t*-BuOOH-treated (75 μM) rat brain mitochondria. *P < 0.05; ***P < 0.001 vs control (nontreated mitochondria). ^+^ *P* < 0.05; ^+2^ *P* < 0.01 vs control (*t*-BuOOH).

Similarly, in microsomes subjected to Fe^2+^/ascorbate-induced lipid peroxidation (which elevated MDA by 250%), both derivatives exhibited robust, concentration-dependent antioxidant profiles. At 10 μM, **1** and **5** decreased MDA formation by 66% and 57%, respectively, maintaining significant protective effects down to 1 μM (Figure 6).

**Figure 6:**
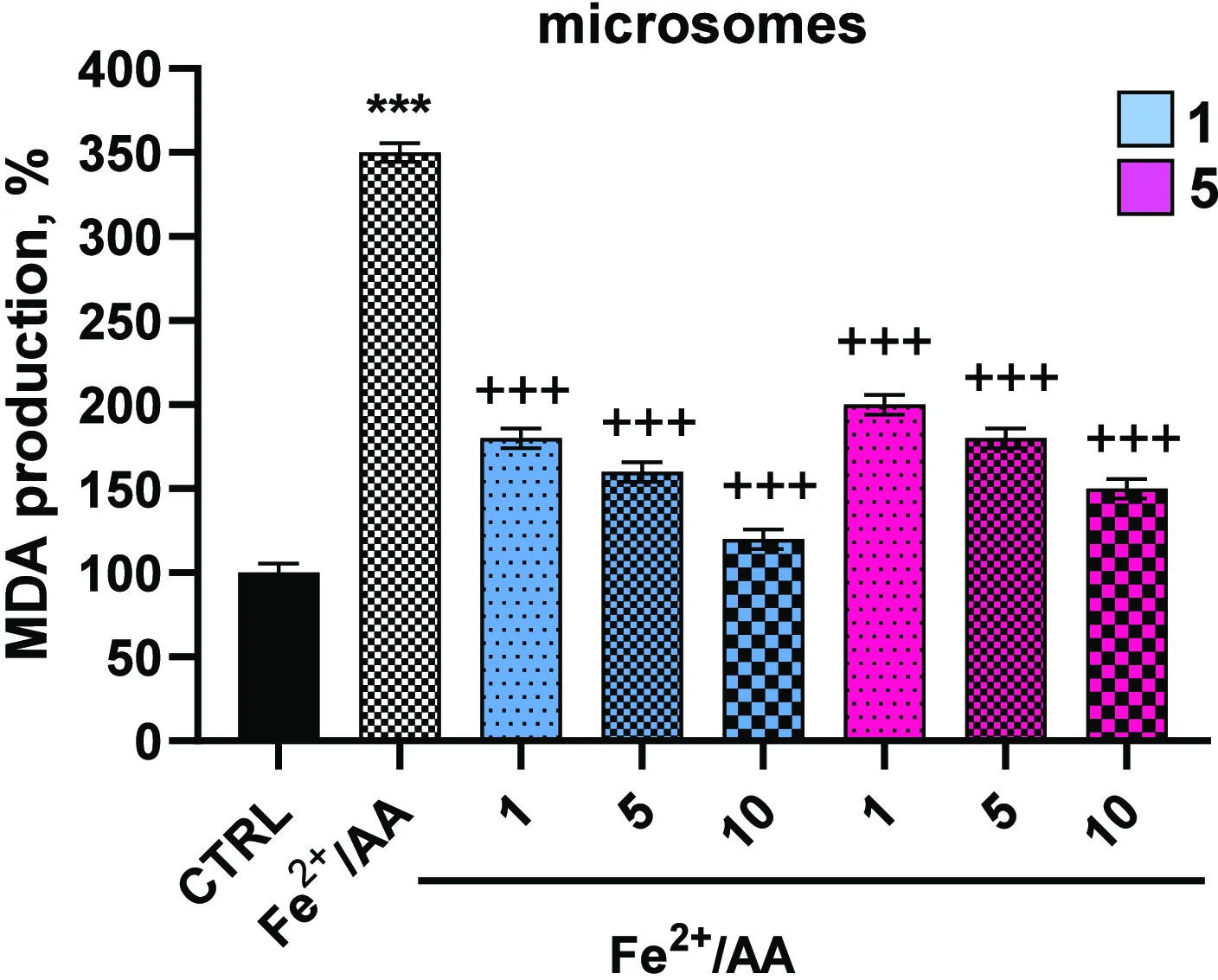
Antioxidant effects of **1** and **5** (1–10 μM) against Fe^2+^/ ascorbate-induced lipid peroxidation in rat brain microsomes. *P < 0.05; ***P < 0.001 vs control (nontreated microsomes). ^+++^ *P* < 0.001 vs control (Fe^2+^/AA).

The compounds’ influence on iron-mediated oxidative degradation of deoxyribose was subsequently assessed to check for secondary redox liabilities. Parental hit **1** exhibited a dose-dependent pro-oxidant effect, enhancing deoxyribose degradation from +3% at 10 μM up to 126.8% of the control at 100 μM (Figure 7). Conversely, the simplified analogue **5** demonstrated a negligible influence across the entire concentration range, showing a baseline 2% reduction at the highest dose (Figure 7). This indicates that molecular truncation favorably suppressed the iron-mediated pro-oxidant liability of the parent scaffold.

**Figure 7:**
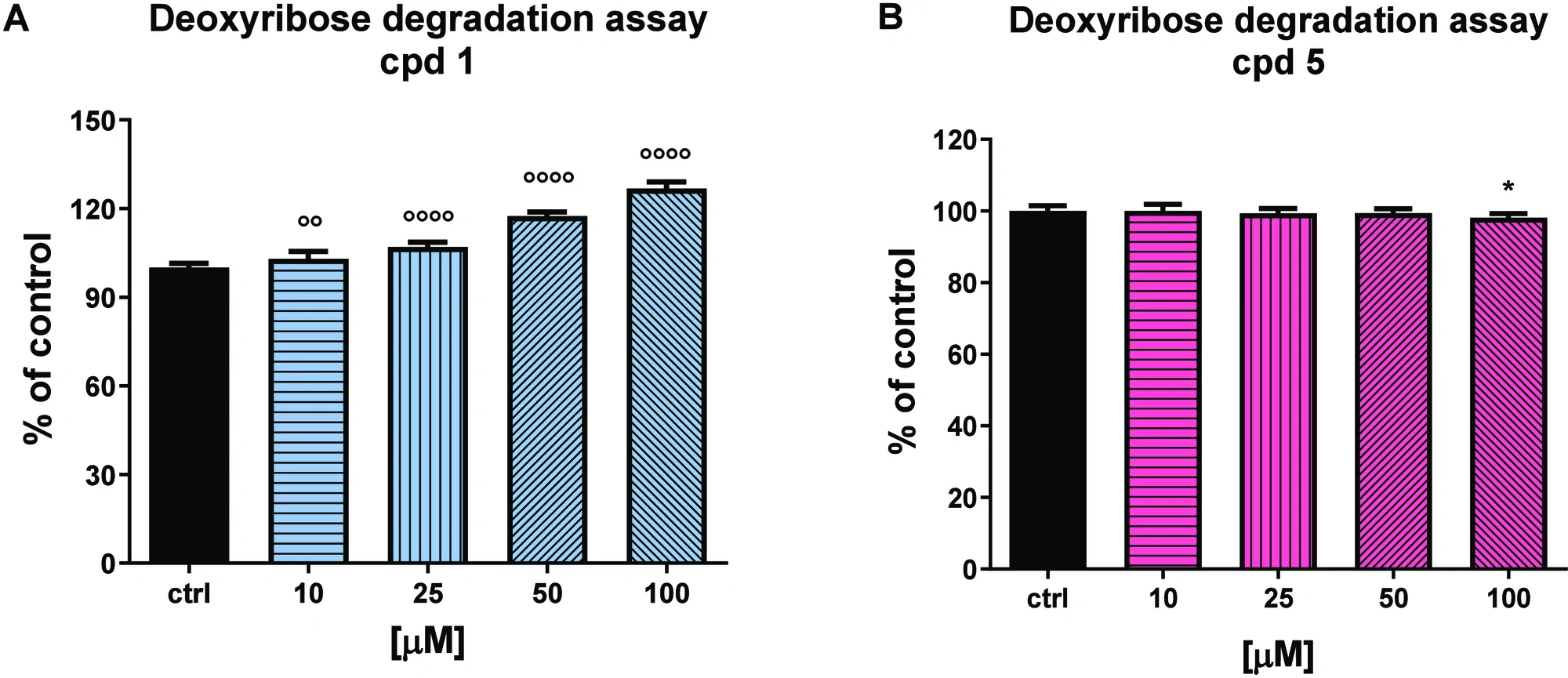
Concentration-dependent effect of the tested derivatives on iron-induced deoxyribose oxidative degradation. Data were analyzed using one-way ANOVA followed by Dunnett’s post hoc test. The significance was set at *p* ≤ 0.05. Significant decreases are indicated as asterisk (*) and significant increases by open circle (°) – */° *p* ≤ 0.05, **/°° *p* ≤ 0.01 and ****/°°°° *p* ≤ 0.0001.

To dissect whether the biological outcomes stemmed from direct NOX enzyme inhibition or secondary radical-scavenging mechanisms, both derivatives were cross-screened in luminol-enhanced chemiluminescence (CL) assays using KO_2_-generated superoxide anion radicals (O_2_^•–^) and hypochlorite ions (ClO^–^) (Figure 8).

**Figure 8:**
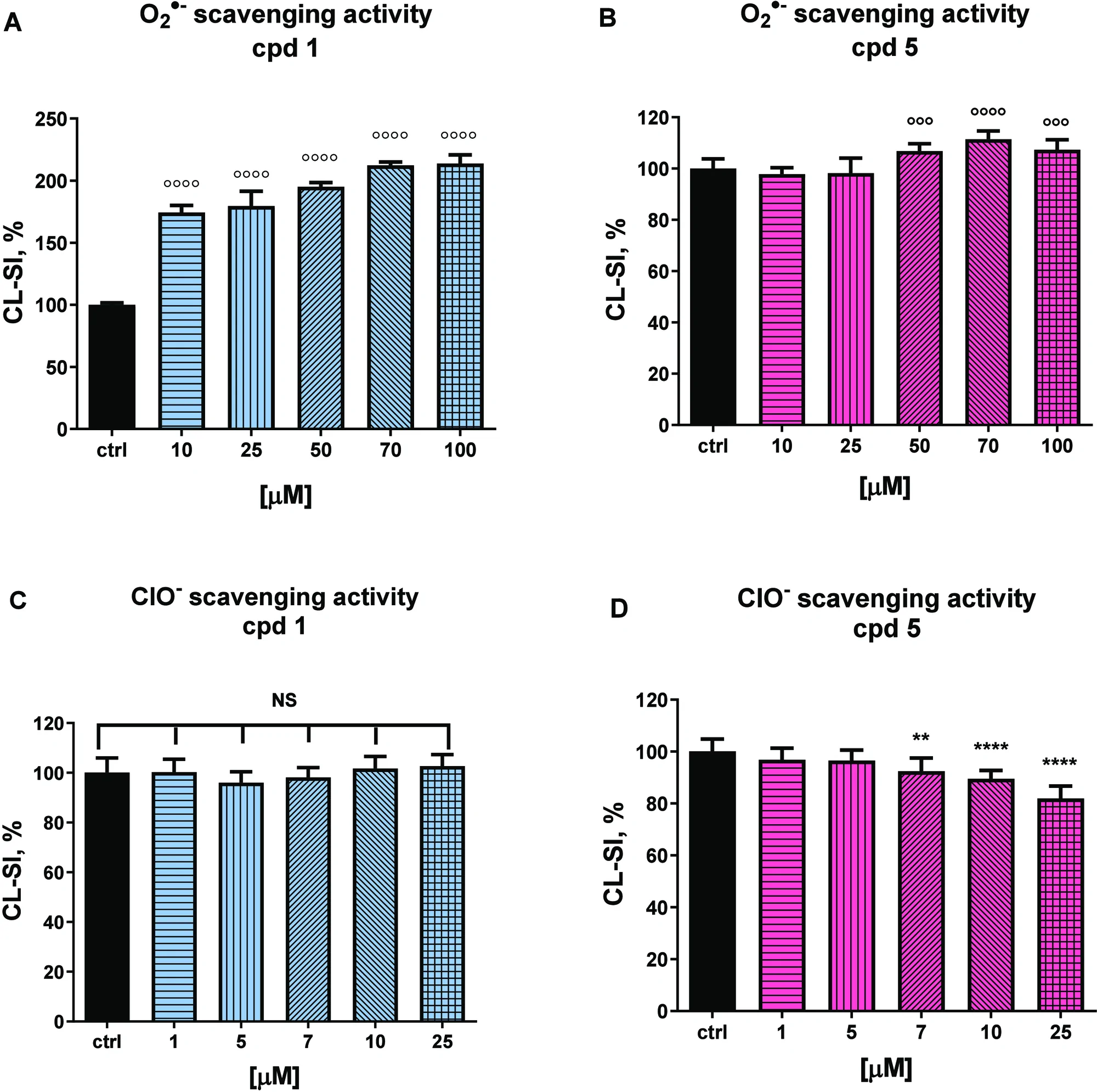
Concentration-dependent effect of the tested derivatives in chemiluminescent model systems: (A and B) System of KO_2_-induced superoxide anion radical generation; (C and D) NaClO-induced hypochlorite ion generation. Results are expressed as mean ± SD of three independent replicates. Statistical significance was evaluated using one-way ANOVA followed by Dunnett’s post hoc test, with significance level set at *p* ≤ 0.05. Significantly lower or higher values were marked with * and ° respectively (NS – not significant; **/°° *p* ≤ 0.01, ***/°°° *p* ≤ 0.001 and ****/°°°° *p* ≤ 0.0001).

Consistent with the deoxyribose data, **1** markedly enhanced the O_2_^•–^ CL signal (+50% at 10 μM; 2-fold at 25 μM), signaling a structural propensity to drive oxidative processes (Figure 8A). Structural optimization successfully resolved this artifact; **5** exerted no stimulation at 10–25 μM and only a minor 11% increase at higher doses (Figure 8B), confirming improved mechanistic specificity and avoiding interference with physiological ROS signaling. In the ClO^–^ system, **1** remained inert, while **5** induced a modest, concentration-dependent signal reduction up to 20% at 25 μM (Figure 8C,D).

To completely rule out assay interference from direct, nonspecific radical quenching, horseradish peroxidase (HRP)-catalyzed oxidation of 2,2′-azino-bis­(3-ethylbenzothiazoline-6-sulfonic acid (ABTS) by H_2_O_2_ was examined alongside a direct ABTS^•+^ scavenging control (Figure 9). Neither compound demonstrated intrinsic radical scavenging capacity against preformed ABTS^•+^ (Figure 9C,D; 99.78% for **1** and 101.28% for **5** at 25 μM). In the complete HRP/H_2_O_2_ enzymatic system, **1** triggered a minor, concentration-dependent decrease (5% at 25 μM), suggesting marginal interaction with H_2_O_2_ (Figure 9A), whereas **5** was totally inactive (Figure 9B). These chemical matrices collectively confirm that the structural optimization yielding **5** successfully stripped away the complex redox liabilities and pro-oxidant tendencies of the parental hit.

**Figure 9:**
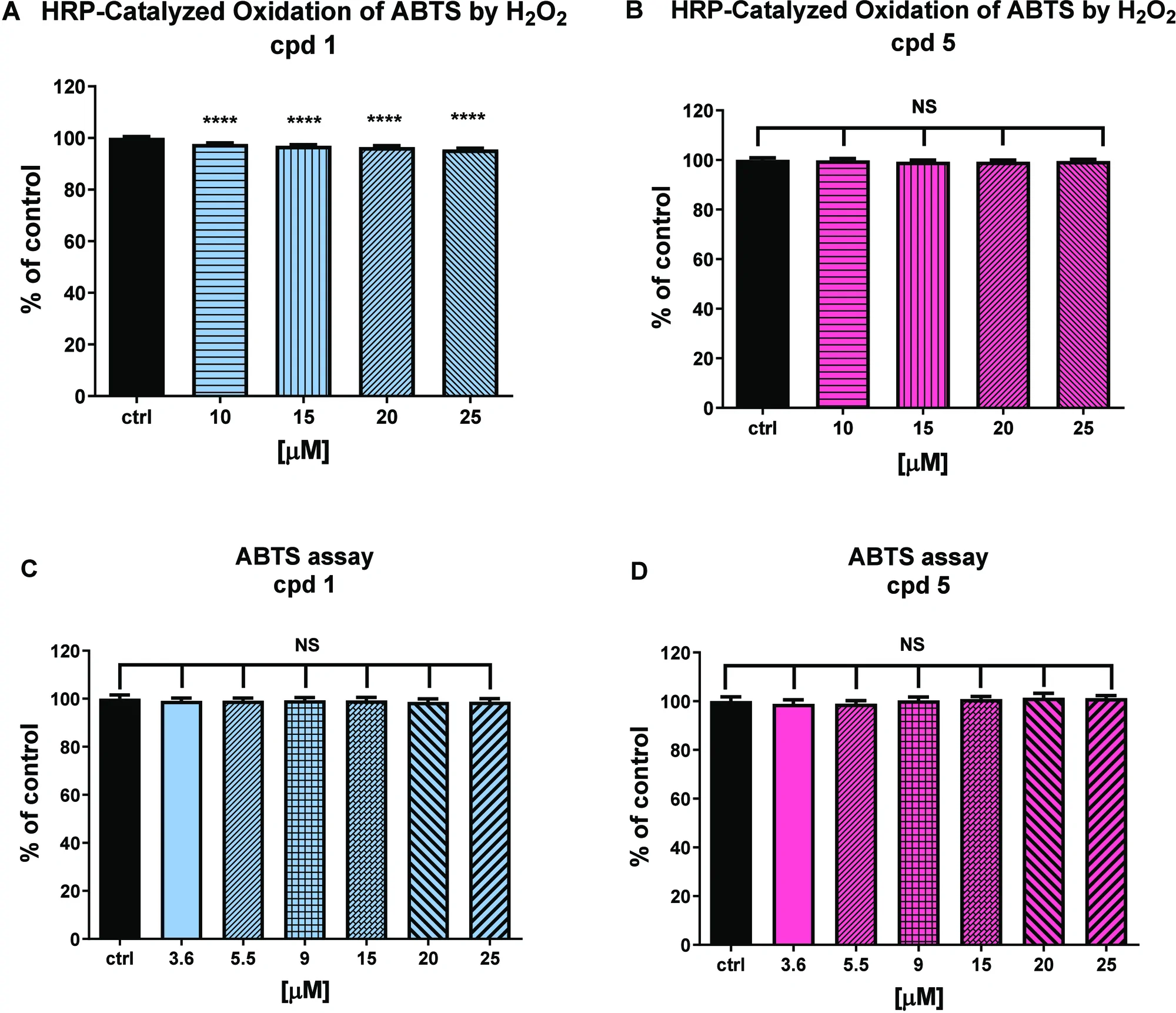
Concentration dependence of the HRP-Catalyzed Oxidation of ABTS by H_2_O_2_ in the presence of the tested NOX inhibitors (A and B) and control assessment of direct ABTS^●+^ radical scavenging as a potential interference (C and D). Data are reported as means ± SD from three independent measurements. Statistical significance was determined using one-way ANOVA (Dunnett’s test). Significance level was set at *p* ≤ 0.05. Significantly lower or higher values were marked with * and ° respectively (NS – not significant; and ****/°°°° *p* ≤ 0.0001).

## Antiproliferative Potential in Cancer Cell Lines

To explore the broader pathological relevance of NOX-dependent redox signaling in oncology, **1** and **5** were screened against a 10-dose panel of human cancer lines selected for distinct NOX expression profiles: Caco-2 (colorectal, NOX1-rich), SU-DHL-6 (lymphoma, NOX2-rich), RPMI-8226 (myeloma, NOX2-rich), HT-1080 (fibrosarcoma, NOX4-rich), and PSN1 (pancreatic, NOX5-rich) (Table 3).

**Table 3:** Antiproliferative Effect of Compounds **1** and **5** in a Panel of Cancer Cells

	Antiproliferative activity, IC_50_, μM				
compd	Caco-2 colorectal cancer	HT-1080 sarcoma	PSN1 pancreatic cancer	RPMI-8226 myeloma	SU-DHL-6 lymphoma
**1**	89.7	31.3	44.9	18.0	13.8
**5**	>200	71.7	185	81.5	60.1

Compound **1** demonstrated its most potent antiproliferative activity against the hematological lines SU-DHL-6 and RPMI-8226, yielding IC_50_ values of 13.8 and 18.0 μM, respectively (Table 3). This alignment strongly correlates with its preferential biochemical inhibition of NOX2 (IC_50_ = 16.7 μM, Table 2). Against solid tumor lines, **1** displayed 2- to 6-fold lower potencies. In stark contrast, the simplified analogue **5** exhibited a complete lack of antiproliferative activity across the entire panel, with IC_50_ values exceeding 60 μM in all cases (Table 3). This sharp drop in cellular efficacy reflects the poor aqueous solubility of **5**, which prevents the compound from reaching the high intracellular concentrations required to achieve effective maximal NOX inhibition, despite its promising cell-free biochemical IC_50_ parameters (Table 2). Antiproliferative curves relative to **1** and **5** in the tested cell lines are reported in Figure S4 in Supporting Information.

## Conclusions

This study establishes clear, final structure–activity relationships for a novel series of [1,3,4]­thiadiazolo­[3,2-*a*]­pyrimidine derivatives designed via a molecular striptease strategy. Extensive truncation of the heterocyclic core or removal of the peripheral 2-phenoxymethyl group (as in **6** and **7**) severely compromised NOX inhibition, underscoring the necessity of extended aromatic interactions within the dehydrogenase domain pocket. However, strategic optimization led to compound **5**, which bears a 4-methoxyphenyl group at the C2 position and a benzylidene motif at the C6 position. Compound **5** successfully retained the parental biochemical inhibitory profile against NOX2, NOX4, and DUOX1, while displaying an improved low-micromolar potency against NOX1 (IC_50_ = 9.0 μM) and NOX5 (IC_50_ = 11.3 μM), despite a low *I*_*max*_.

The promising intrinsic neuroprotective profiles of **1** and **5** were confirmed in rat brain synaptosomes, mitochondria, and microsomes, where they effectively countered oxidative stress and lipid peroxidation at low micromolar concentrations. Crucially, in-depth evaluation across multiple ROS-generating chemical assays revealed that the structural simplification of **1** into **5** effectively eliminated the pro-oxidant liabilities and complex radical-scavenging artifacts inherent to the parent molecule, ensuring high mechanistic specificity. While **1** stands as a robust chemical probe with clear therapeutic potential against hematological malignancies, **5** represents a highly streamlined, clean chemotype. To overcome the physicochemical limitations of **5**, future optimization efforts will combine prodrug strategies or hydrophilic formulations with rational structure-based modifications. Based on our docking analyses within the NADPH binding domain, key binding interactions are localized around the core scaffold, leaving vector positions on the outer rings oriented toward solvent-exposed regions. Consequently, the aqueous solubility of **5** can be systematically improved without disrupting crucial target engagements by (i) replacing the phenyl ring of the benzylidene moiety with basic nitrogen-containing heterocycles (such as pyridine or morpholine) or adding polar hydroxyl groups; (ii) modifying the methoxyphenyl ring with hydrophilic solubilizing handles, such as hydroxy group, short PEG-like chains or terminal aliphatic amines; or (iii) employing bioreversible prodrug strategies (e.g., phosphate or ester derivatives). Additionally, as **5** features an α/β-unsaturated system with theoretical Michael acceptor character, future optimization rounds will include thiol-reactivity assays (e.g., GSH binding) to confirm nonpromiscuous, reversible target interactions and rule out unspecific covalent labeling.

Once these solubility/reactivity bottlenecks are addressed, the scaffold of **5** can represent a highly promising, artifact-free architectural starting point for the development of potent, isoform-selective NOX inhibitors.

## Supplementary Material



## Notes

Experimental Section: Synthetic procedures and analytical data of selected compounds, biochemical evaluation, computational methods, neuroprotective, antioxidant, and radical scavenging assays, anticancer assays. Inhibition curves for NOXs inhibition. Antiproliferative curves for tested cancer cell lines treated with 1 and 5 (PDF)

## Abbreviations

ABTS: 2,2′-azino-bis­(3-ethylbenzothiazoline-6-sulfonic acid
ANOVA: One-way analysis of variance
apo: apoprotein
*t*-BuOOH: *tert*-butyl hydroperoxide
CL: chemiluminescence
Cryo-EM: cryo-electron microscopy
*cs*: *Cylindrospermum stagnale*
DHD: dehydrogenase domain
DUOX: dual oxidase
Fe^2+^/AA: Fe^2+^/ascorbate
GSH: reduced glutathione
HRP: horseradish peroxidase
*I*_*max*_: maximum inhibition
KO_2_: potassium superoxide
MPP^+^: 1-methyl-4-phenylpyridinium
MTT: 3-[4,5-dimethylthiazol-2-yl]-2,5-diphenyltetrazolium bromide
NaClO: sodium hypochlorite
NOX: NADPH oxidase
O_2_^●–^: superoxide radicals
6-OHDA: 6-hydroxydopamine
