# Supplementary material for: Molecular Striptease of a Thiadiazolopyrimidine Hit Yields a Streamlined, Artifact-Free NADPH Mimic for NOX Inhibition
Source: ACS Med Chem Lett. 2026 Aug 8;17(9):2045–54. doi: 10.1021/acsmedchemlett.6c00370 (PMC13573257; doi:10.1021/acsmedchemlett.6c00370)
Supplement: Supplementary file 1 [file ml6c00370_si_001.pdf]

Emanuele Fabbrizi,[†] Andrea Mancini,[†] Chiara Lambona, Salvatore Macrì, Marianna Nalli, Francesco Quilli, Federico Pandolfi, Domenico Raimondo, Sara Marchese, Andrea Mattevi, Jihyeon Lee, Minhye Choi, Yun Soo Bae, Nadya Hristova-Avakumova, Magdalena Kondeva-Burdina, Denitsa Stefanova, Neda Anastassova, Sergio Valente, Dante Rotili, Antonello Mai[*]

Molecular Striptease of a Thiadiazolopyrimidine Hit Yields a Streamlined, Artifact-Free NADPH Mimic for NOX Inhibition

Supporting Information

Content:

**Experimental Section**	p. S3
**Chemistry**	p. S3
**Table S1.** Elemental Analysis for Final Compounds **1-7**.	p. S7
**^{1}H NMR and ESI-MS Spectra of Final Compounds 1–7**	p. S8
**Biochemistry**	p. S15
**Computational procedures**	p. S16
**Neuroprotection Assays**	p. S17
**Antioxidant/ROS Scavenging Activity**	p. S23
**Anticancer Assays**	p. S24
**Figure S1. Inhibition curves for the NOX inhibitors 1, 2, and 5**	p. S26
**Figure S2. Stability assays: HPLC traces of compound 1**	p. S27
**Figure S3. *In vitro* cytotoxicity evaluation of compound 1 (MC5328) and compound 5 (MC5335) on SH-SY5Y cells**	p. S28
**Figure S4. Antiproliferative curves for compounds 1 and 5**	p. S29
**References**	p. S30

Experimental Section

Chemistry

Melting points were determined on a Buchi 530 melting point apparatus. ^{1}H NMR spectra were recorded at 400 MHz on a Bruker AC 400 spectrometer; chemical shifts are reported in δ (ppm) units relative to the internal reference tetramethyl silane (Me_4Si). Low-resolution mass spectra of final and intermediate compounds were recorded on a MSQ Plus Mass Spectrometer (Thermo Fisher), samples were injected by a Harvard pump using a flow rate of 100 µL/min, infused in the Electrospray system. All compounds were routinely checked by TLC, ^{1}H NMR spectra and MS. TLC analyses were performed on aluminum-backed silica gel plates (Merck DC, Alufolien Kieselgel 60 F254) with spots visualized by UV light. All solvents were reagent grade and, when necessary, were purified and dried by standard methods. Concentration of solutions after reactions and extractions involved using a rotary evaporator operating at a reduced pressure of ca. 20 Torr. Organic solutions were dried over anhydrous sodium sulfate. Elemental analysis has been used to determine the purity of the described compounds, which is >95 %. Analytical results are within ±0.40% of the theoretical values (Table S1, pag S6). All chemicals were purchased from Sigma Aldrich, Milan (Italy), BLD-Pharma, Kaiserslautern (Germany), or TCI Europe N.V., Zwijndrecht (Belgium), and were of the highest purity.

General Procedure for the synthesis of 2-substituted-6-benzylidene-[1,3,4]thiadiazolo[3,2-a]pyrimidine derivatives (1-6). Example: 3-((5-imino-7-oxo-2-(phenoxymethyl)-5*H*-[1,3,4]thiadiazolo[3,2-*a*]pyrimidin-6(7*H*)-ylidene)methyl)phenyl 3-Methylbenzoate (1, MC5328). To a stirred solution of intermediate **9** (60 mg, 0.219 mmol, 1 equiv) in anhydrous DMF (0.5 mL) and dry ethanol (0.5 mL) was added 3-formylphenyl 3-methylbenzoate (55.2 mg, 0.23 mmol, 1.05 equiv), followed by triethylamine (3 drops, catalytic amount). The reaction mixture was heated at 50 °C for 3 h. After cooling to room temperature, the mixture was poured into water and extracted with ethyl acetate. The combined organic layers were washed with brine, dried over anhydrous Na_2SO_4, filtered, and

concentrated under reduced pressure. The crude product was purified by silica gel column chromatography ($CHCl_3$/MeOH, 1:1 %v/v) to afford the target compound **1** as a white solid. Mp: 233-234 °C. Yield: 89%; rec. solv.: ethanol. ^{1}H NMR (400 MHz, DMSO-d_6) δ 2.44 (s, 3H, -C*H_3*), 5.50 (s, 2H, Ph-OC*H_2*-), 7.02 (t, 1H, benzene ring), 7.09 (d, 2H, benzene ring), 7.35 (t, 2H, benzene ring), 7.53 (t, 1H, benzene ring), 7.59 (t, 2H, benzene ring), 7.72 (t, 1H, benzene ring), 7.92 (s, 1H, benzene ring), 7.98-8.07 (m, 3H, benzene ring), 8.51 (s, 1H, -C*H*= benzylidene) 13.86 (*br* s, 1H, N*H*). MS (ESI), m/z: 495 $[M-H]^-$.

3-((5-Imino-7-oxo-5*H*-[1,3,4]thiadiazolo[3,2-*a*]pyrimidin-6(7*H*)-ylidene)methyl)phenyl 3-Methylbenzoate (2, MC5333). White solid. Mp: 231-232 °C. Yield: 81%; rec. solv.: ethanol. ^{1}H NMR (400 MHz, DMSO-d_6) δ 2.44 (s, 3H, -C*H_3*), 7.41 (t, 1H, benzene ring), 7.57-7.60 (m, 2H, benzene ring), 7.71 (t, 2H, benzene ring), 7.93 (s, 1H, benzene ring), 7.97-8.00 (m, 2H, benzene ring), 8.52 (s, 1H, thiadiazole ring), 9.15 (s, 1H, -C*H*= benzylidene) 13.85 (*br* s, 1H, N*H*). MS (ESI), m/z: 389 $[M-H]^-$.

5-Imino-6-(3-methoxybenzylidene)-2-(phenoxymethyl)-5,6-dihydro-7*H*-[1,3,4]thiadiazolo [3,2-*a*]pyrimidin-7-one (3, MC5329). White solid. Mp: 219-221 °C. Yield: 85%; rec. solv.: ethanol. ^{1}H NMR (400 MHz, DMSO-d_6) δ 3.84 (s, 3H, -OC*H_3*), 5.69 (s, 2H, Ph-OC*H_2*-), 7.02 (t, 1H, benzene ring), 7.09 (d, 2H, benzene ring), 7.22 (d, 1H, benzene ring), 7.35 (t, 2H, benzene ring), 7.53 (t, 1H, benzene ring), 7.59-7.62 (m, 2H, benzene ring), 8.47 (s, 1H, -C*H*= benzylidene) 14.01 (*br* s, 1H, N*H*). MS (ESI), m/z: 391 $[M-H]^-$.

6-Benzylidene-5-imino-2-(phenoxymethyl)-5,6-dihydro-7*H*-[1,3,4]thiadiazolo[3,2-*a*]pyrimidin-7-one (4, MC5330). White solid. Mp: 209-210 °C. Yield: 77%; rec. solv.: ethanol. ^{1}H NMR (400 MHz, DMSO-d_6) δ 5.50 (s, 2H, Ph-OC*H_2*-), 7.02 (t, 1H, benzene ring), 7.09 (d, 2H, benzene ring), 7.35 (t, 2H, benzene ring), 7.61-7.63 (m, 3H, benzene ring), 8.02 (d, 2H, benzene ring), 8.50 (s, 1H, -C*H*= benzylidene) 13.88 (*br* s, 1H, N*H*). MS (ESI), m/z: 361 $[M-H]^-$.

6-Benzylidene-5-imino-2-(4-methoxyphenyl)-5,6-dihydro-7*H*-[1,3,4]thiadiazolo[3,2-*a*]pyrimidin-7-one (5, MC5335). Pale yellow solid. Mp: 227-228 °C. Yield: 79%; rec. solv.: ethanol. ^{1}H NMR (400 MHz, DMSO-d$_6$) δ 3.85 (s, 3H, -OC*H$_3$*), 7.11 (d, 2H, benzene ring), 7.61-7.64 (m, 3H, benzene ring), 7.90 (d, 2H, benzene ring), 8.04 (d, 2H, benzene ring), 8.62 (s, 1H, -C*H*= benzylidene) 14.35 (*br* s, 1H, N*H*). MS (ESI), m/z: 361 [M-H]$^-$.

6-Benzylidene-5-imino-5,6-dihydro-7*H*-[1,3,4]thiadiazolo[3,2-*a*]pyrimidin-7-one (6, MC5334). Pale yellow solid. Mp: 241-242 °C. Yield: 83%; rec. solv.: ethanol. ^{1}H NMR (400 MHz, DMSO-d$_6$) δ 7.47-7.63 (m, 3H, benzene ring), 8.03 (d, 2H, benzene ring), 8.51 (s, 1H, thiadiazole ring), 9.16 (s, 1H, -C*H*= benzylidene), 13.99 (*br* s, 1H, N*H*). MS (ESI), m/z: 255 [M-H]$^-$.

General Procedure for the Synthesis of 5-Imino-5,6-dihydro-7*H*-[1,3,4]thiadiazolo[3,2-*a*]pyrimidin-7-ones (7-9). Example: 5-Imino-2-(phenoxymethyl)-5,6-dihydro-7*H*-[1,3,4]thiadiazolo[3,2-*a*]pyrimidin-7-one (9, MC5327). Sodium metal (77.6 mg, 3.38 mmol, 1 equiv) was dissolved in anhydrous ethanol (11.2 mL) under stirring. 5-(Phenoxymethyl)-1,3,4-thiadiazol-2-amine (700 mg, 3.38 mmol, 1 equiv) and ethyl cyanoacetate (0.361 ml, 382.2 mg, 3.38 mmol, 1 equiv) were added sequentially to the resulting solution. The reaction mixture was heated at reflux for 2 h, then allowed to cool to room temperature. The mixture was carefully quenched by the dropwise addition of 1N HCl until acidic pH. The resulting precipitate was collected by vacuum filtration, washed with water, and dried under vacuum to afford intermediate **9** as a colorless solid. Mp: 229-230 °C. Yield: 89 %; rec. solv.: ethanol. ^{1}H NMR (400 MHz, DMSO-d$_6$) δ 4.09 (s, 2H, Ph-OC*H$_2$*-), 5.51 (s, 2H, -CO-C*H$_2$*-CNH-), 7.00 (t, 1H, benzene ring), 7.07 (d, 2H, benzene ring), 7.33 (t, 2H, benzene ring), 12.96 (*br* s, 1H, N*H*). MS (ESI), m/z: 273 [M-H]$^-$.

5-Imino-5,6-dihydro-7*H*-[1,3,4]thiadiazolo[3,2-*a*]pyrimidin-7-one (7). Mp: 278-282 °C. Yield: 89 %; rec. solv.: ethanol. ^{1}H NMR (400 MHz, DMSO-d$_6$) δ 4.10 (s,2H, -CO-C*H$_2$*-CNH-), 9.22 (s, 1H, -C*H*- thiadiazole ring), 13.31 (*br* s, 1H, N*H*). MS (ESI), m/z: 167 [M-H]$^-$.

5-Imino-2-(4-methoxyphenyl)-5,6-dihydro-7*H*-[1,3,4]thiadiazolo[3,2-*a*]pyrimidin-7-one (8). Mp: 278-282 °C. Yield: 89 %; rec. solv.: ethanol. ^{1}H NMR (400 MHz, DMSO-d$_6$) δ 3.84 (s, 3H, OCH_3), 4.12 (s, 2H, -CO-CH_2-CNH-), 7.08 (d, 2H, benzene ring), 7.90 (d, 2H, benzene ring), 12.96 (*br* s, 1H, N*H*). MS (ESI), m/z: 273 [M-H]$^-$.

Synthesis of 3-Formylphenyl 3-Methylbenzoate (10). To a stirred solution of 3-hydroxybenzaldehyde (1.0 g, 12.28 mmol, 1.0 equiv) and triethylamine (1.65 g, 2.3 mL, 16.37 mmol, 2.0 equiv) in anhydrous dichloromethane (5 mL) under a nitrogen atmosphere, a solution of 3-methylbenzoyl chloride (1.79 g, 1.5 mL, 12.28 mmol, 1.5 equiv) in anhydrous dichloromethane (3 mL) was added dropwise at 0 °C. The reaction mixture was then allowed to warm to room temperature and stirred for 1 h. Reaction progress was monitored by TLC. Upon complete consumption of the starting material, the reaction was quenched by the addition of saturated aqueous $NaHCO_3$, and the mixture was extracted with dichloromethane. The combined organic layers were washed with brine, dried over anhydrous sodium sulfate, filtered, and concentrated under reduced pressure. The crude product was purified by silica gel column chromatography using ethyl acetate/*n*-hexane (1:15, v/v) as the eluent, affording compound **10** as a white solid (yield: 85%). ^{1}H NMR (400 MHz, DMSO-d$_6$) δ 2.37 (s, 3H, -CH_3), 7.25-7.31 (m, 1H, benzene ring), 7.33-7.39 (m, 2H, benzene ring), 7.52 (t, 1H, benzene ring), 7.66-7.69 (dt, 1H, benzene ring), 7.72 (t, 1H, benzene ring), 7.95 (t, 1H, benzene ring), 7.97-8.00 (m, 1H, benzene ring), 9.92 (s, 1H, -C*H*O).

Table S1. Elemental Analysis for Final Compounds **1-7**.

**Elemental Analysis of compounds 1-7**									
	**MW**	**Predicted**				**Calculated**			
		% C	% H	% N	% S	% C	% H	% N	*% S*
**1 (MC5333)**	390.417	61.53	3.61	14.35	8.21	61.66	3.63	14.29	8.19
**2 (MC5334)**	256.283	56.24	3.15	21.86	12.51	56.32	3.17	21.82	12.49
**3 (MC5335)**	362.407	62.97	3.89	15.46	8.85	63.07	3.91	15.41	8.83
**4 (MC5328)**	496.541	65.31	4.06	11.28	6.46	65.43	4.08	11.22	6.45
**5 (MC5329)**	392.433	61.21	4.11	14.28	8.17	61.30	4.13	14.22	8.16
**6 (MC5330)**	362.407	62.97	3.89	15.46	8.85	61.66	3.63	14.31	8.19
**7 (MC5327)**	274.298	52.55	3.67	20.43	11.69	52.65	3.69	20.38	11.67

¹H NMR and ESI-MS Spectra of Final Compounds 1–7

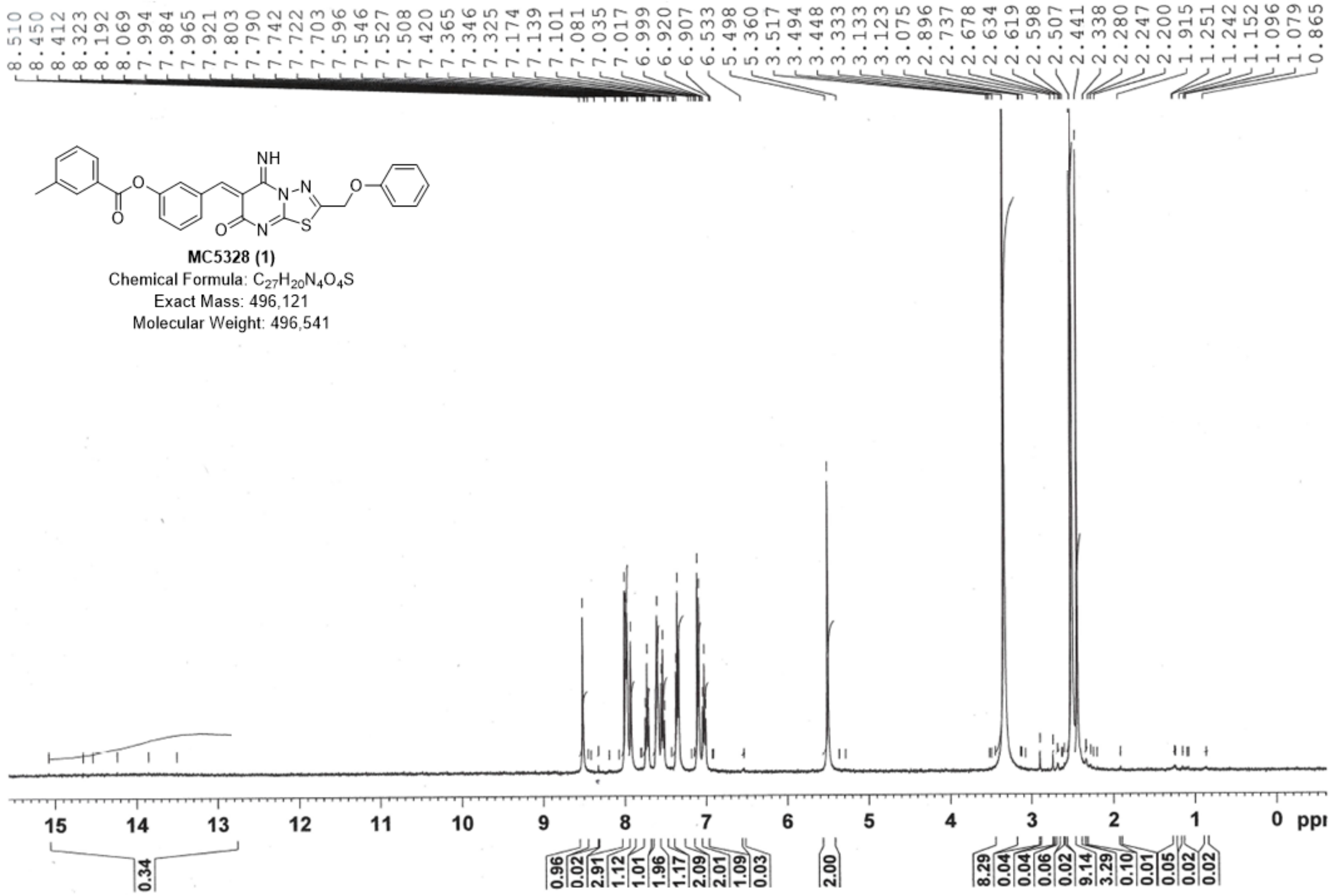

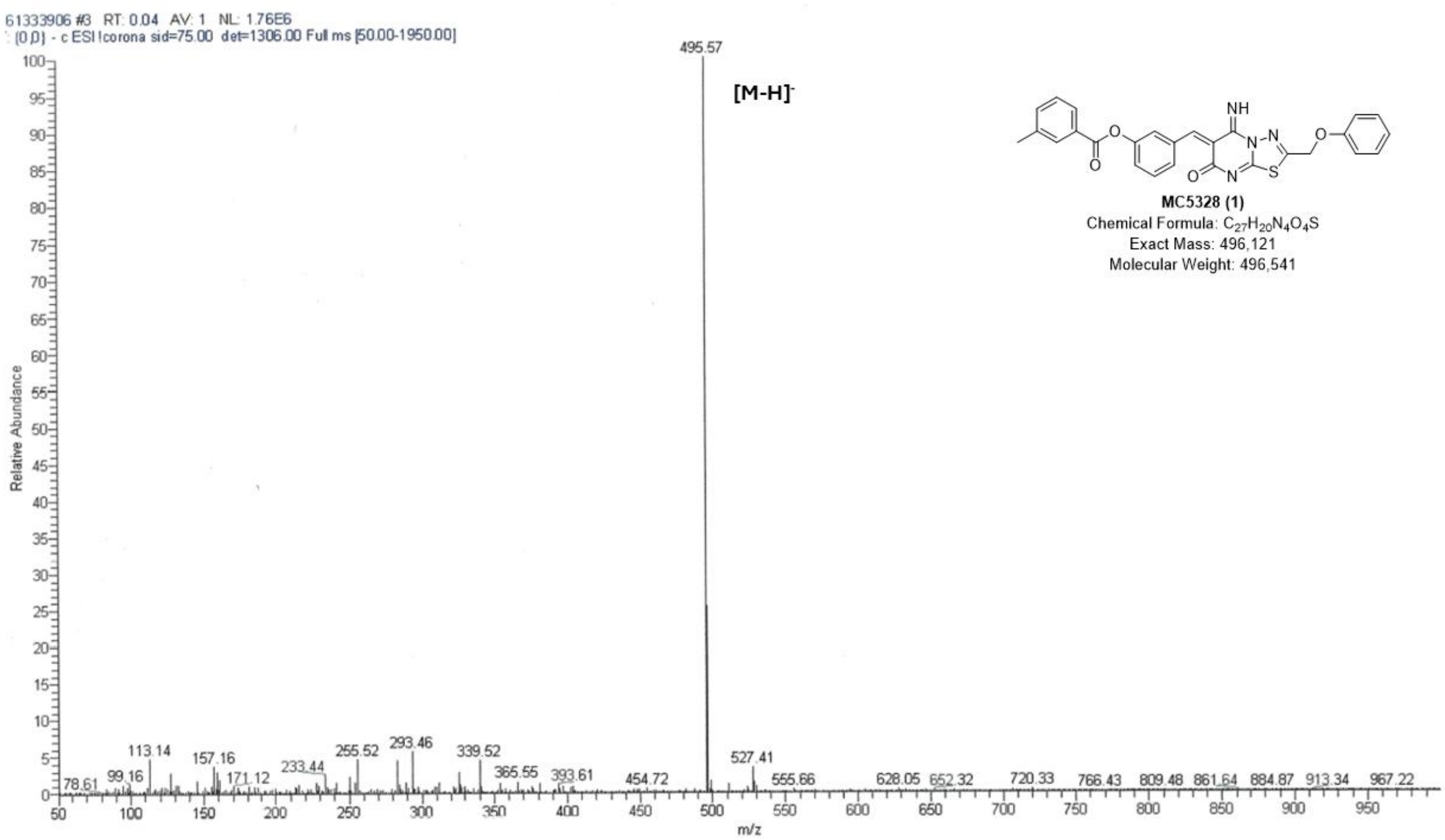

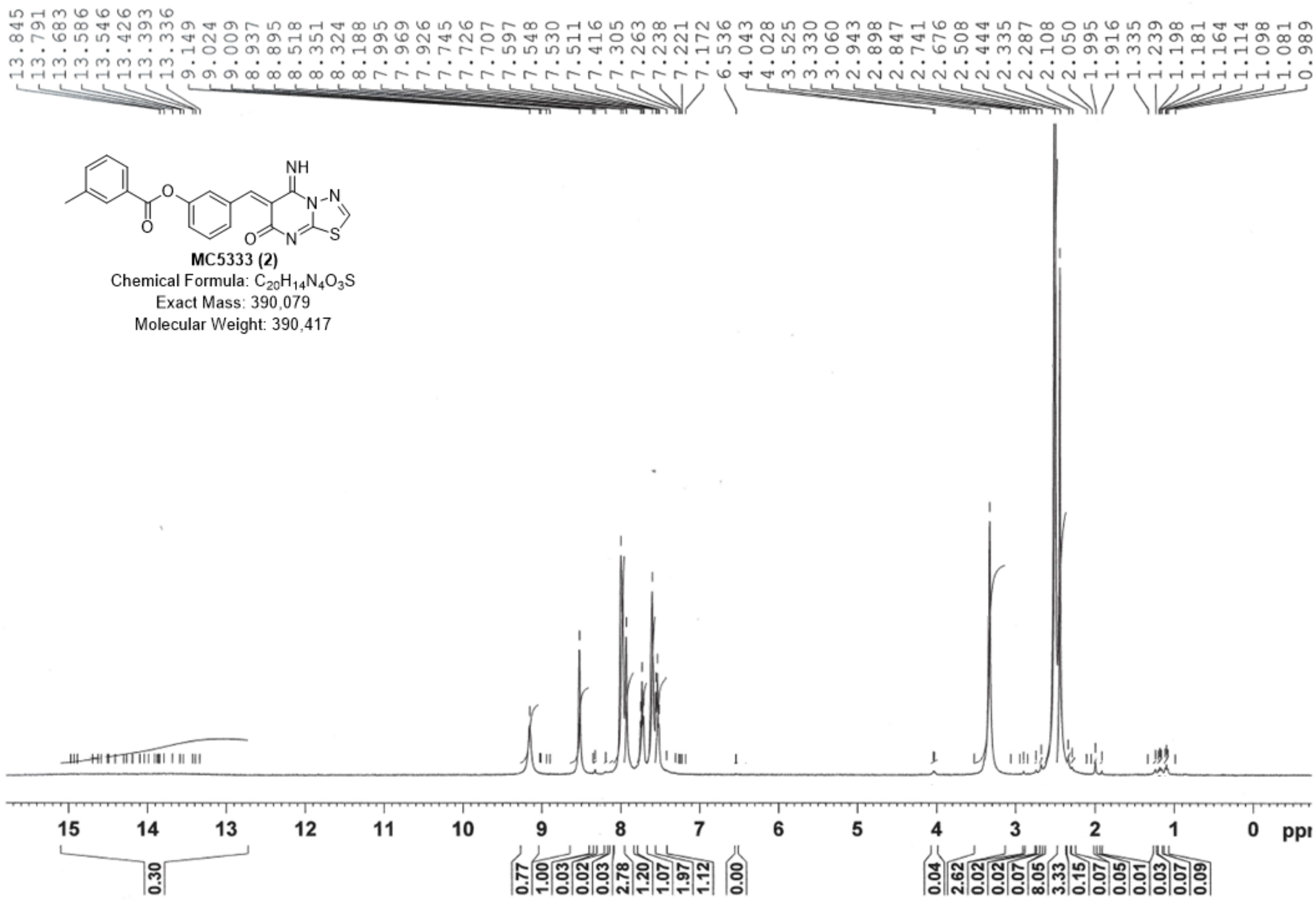
13.845 13.791 13.683 13.586 13.546 13.426 13.393 13.336 9.149 9.024 9.009 8.937 8.895 8.518 8.351 8.324 8.188 7.995 7.969 7.926 7.745 7.726 7.707 7.597 7.548 7.530 7.511 7.416 7.305 7.263 7.238 7.221 7.172 6.536 4.043 4.028 3.525 3.330 3.060 2.943 2.898 2.847 2.741 2.676 2.508 2.444 2.335 2.287 2.108 2.050 1.995 1.916 1.335 1.239 1.198 1.181 1.164 1.114 1.098 1.081 0.989
NH
O
N
S
MC5333 (2)
Chemical Formula: $C_{20}H_{14}N_4O_3S$
Exact Mass: 390,079
Molecular Weight: 390,417
15 14 13 12 11 10 9 8 7 6 5 4 3 2 1 0 ppm
0.30
0.77 1.00 0.03 0.02 0.03 2.78 1.20 1.07 1.97 1.12 0.00
0.04 2.62 0.02 0.02 0.07 8.05 3.33 0.15 0.07 0.05 0.01 0.03 0.07 0.09

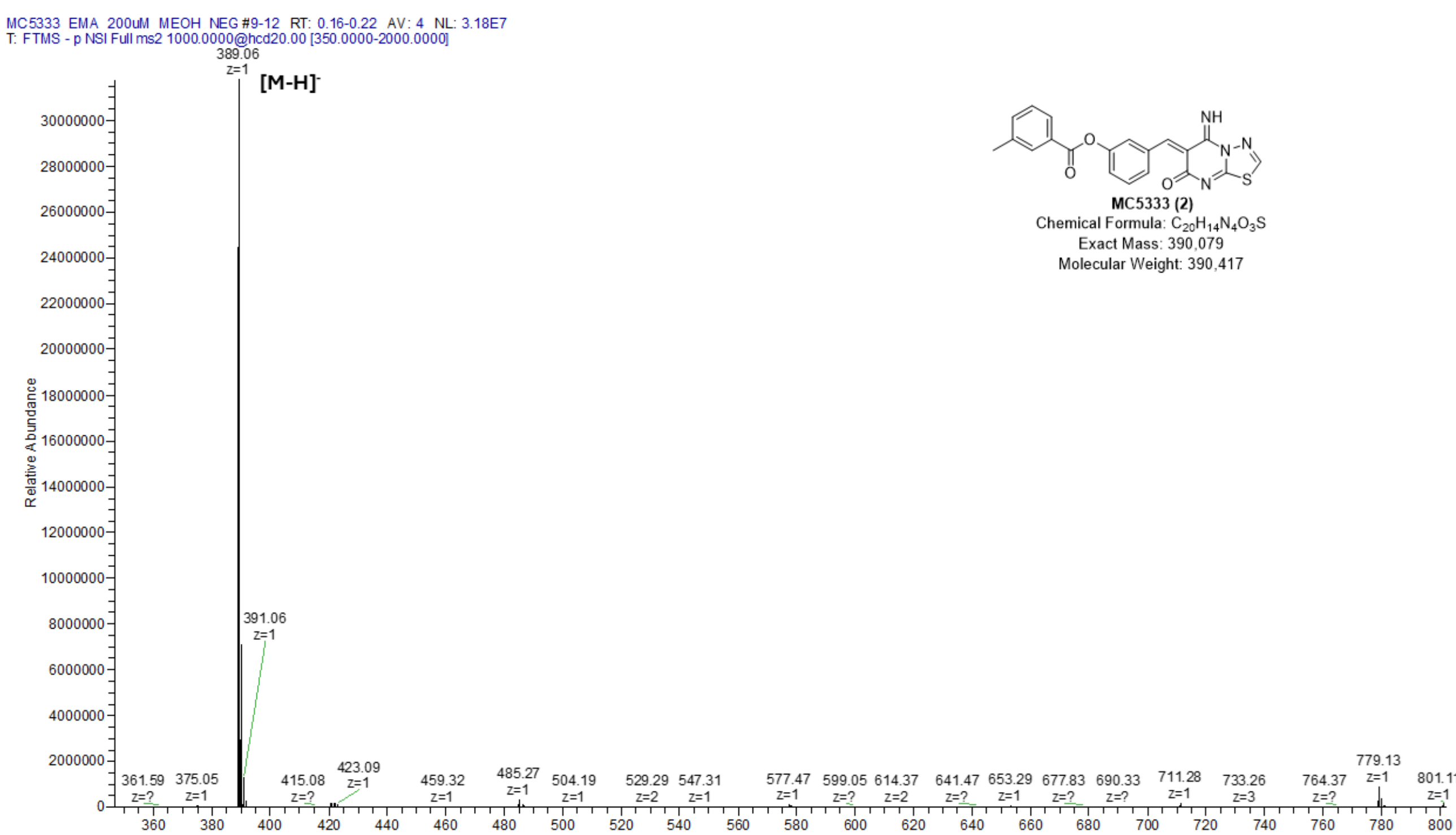
MC5333 EMA 200uM MEOH NEG #9-12 RT: 0.16-0.22 AV: 4 NL: 3.18E7
T: FTMS - p NSI Full ms2 1000.0000@hcd20.00 [350.0000-2000.0000]
389.06 z=1
[M-H]-
391.06 z=1
361.59 z=? 375.05 z=1 415.08 z=? 423.09 z=1 459.32 z=1 485.27 z=1 504.19 z=1 529.29 z=2 547.31 z=1 577.47 z=1 599.05 z=? 614.37 z=2 641.47 z=? 653.29 z=1 677.83 z=? 690.33 z=? 711.28 z=1 733.26 z=3 764.37 z=? 779.13 z=1 801.11 z=1
Relative Abundance
m/z
NH
O
N
S
MC5333 (2)
Chemical Formula: $C_{20}H_{14}N_4O_3S$
Exact Mass: 390,079
Molecular Weight: 390,417

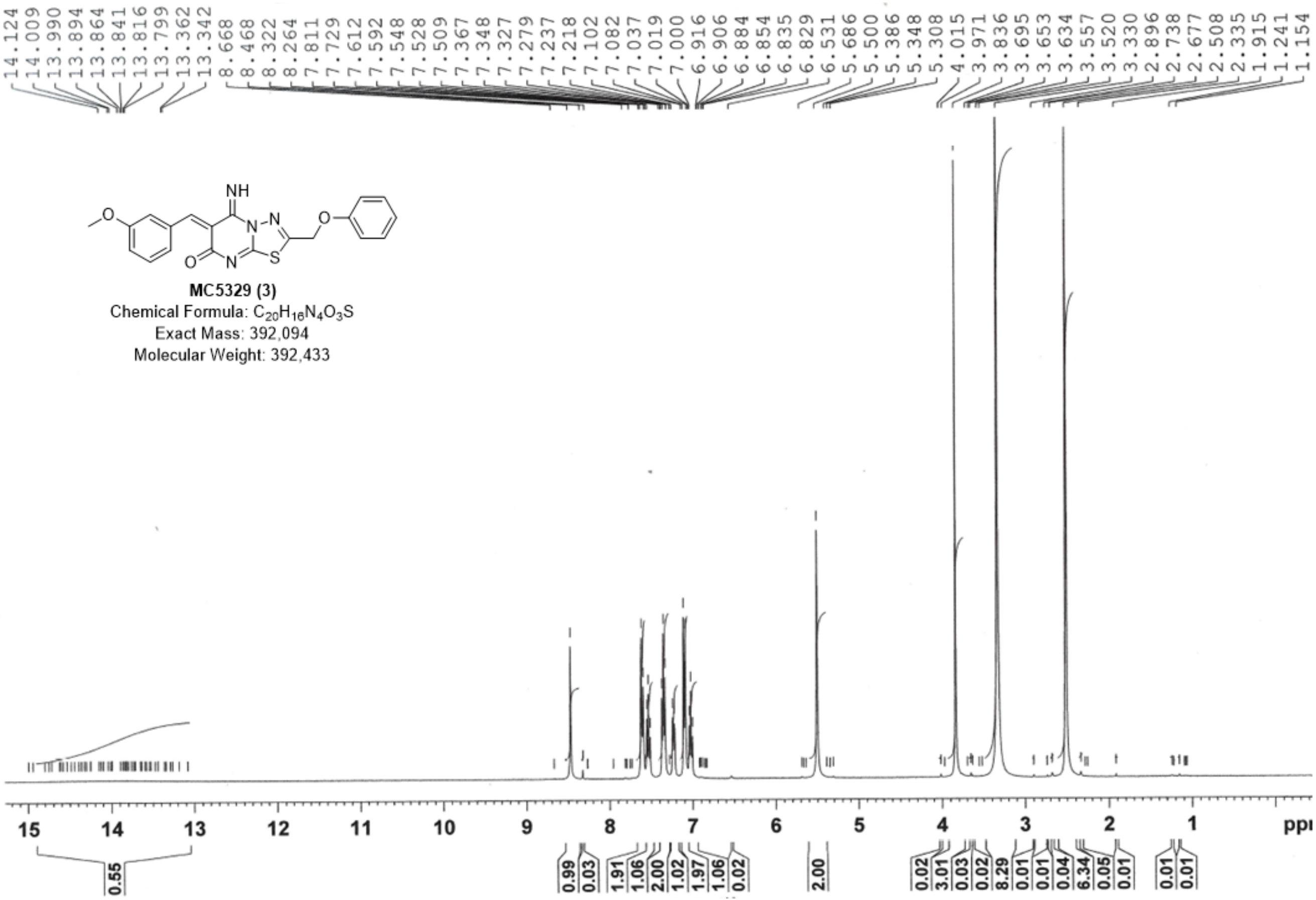
14.124 14.009 13.990 13.894 13.864 13.841 13.816 13.799 13.362 13.342 8.668 8.468 8.322 8.264 7.811 7.729 7.612 7.592 7.548 7.528 7.509 7.367 7.348 7.327 7.279 7.237 7.218 7.102 7.082 7.037 7.019 7.000 6.916 6.906 6.884 6.854 6.835 6.829 6.531 5.686 5.500 5.386 5.348 5.308 4.015 3.971 3.836 3.695 3.653 3.634 3.557 3.520 3.330 2.896 2.738 2.677 2.508 2.335 1.915 1.241 1.154
MC5329 (3)
Chemical Formula: $C_{20}H_{16}N_4O_3S$
Exact Mass: 392,094
Molecular Weight: 392,433
ppm
0.55
0.99 0.03 1.91 1.06 2.00 1.02 1.97 1.06 0.02
2.00
0.02 3.01 0.03 0.02 8.29 0.01 0.01 0.04 6.34 0.05 0.01 0.01 0.01

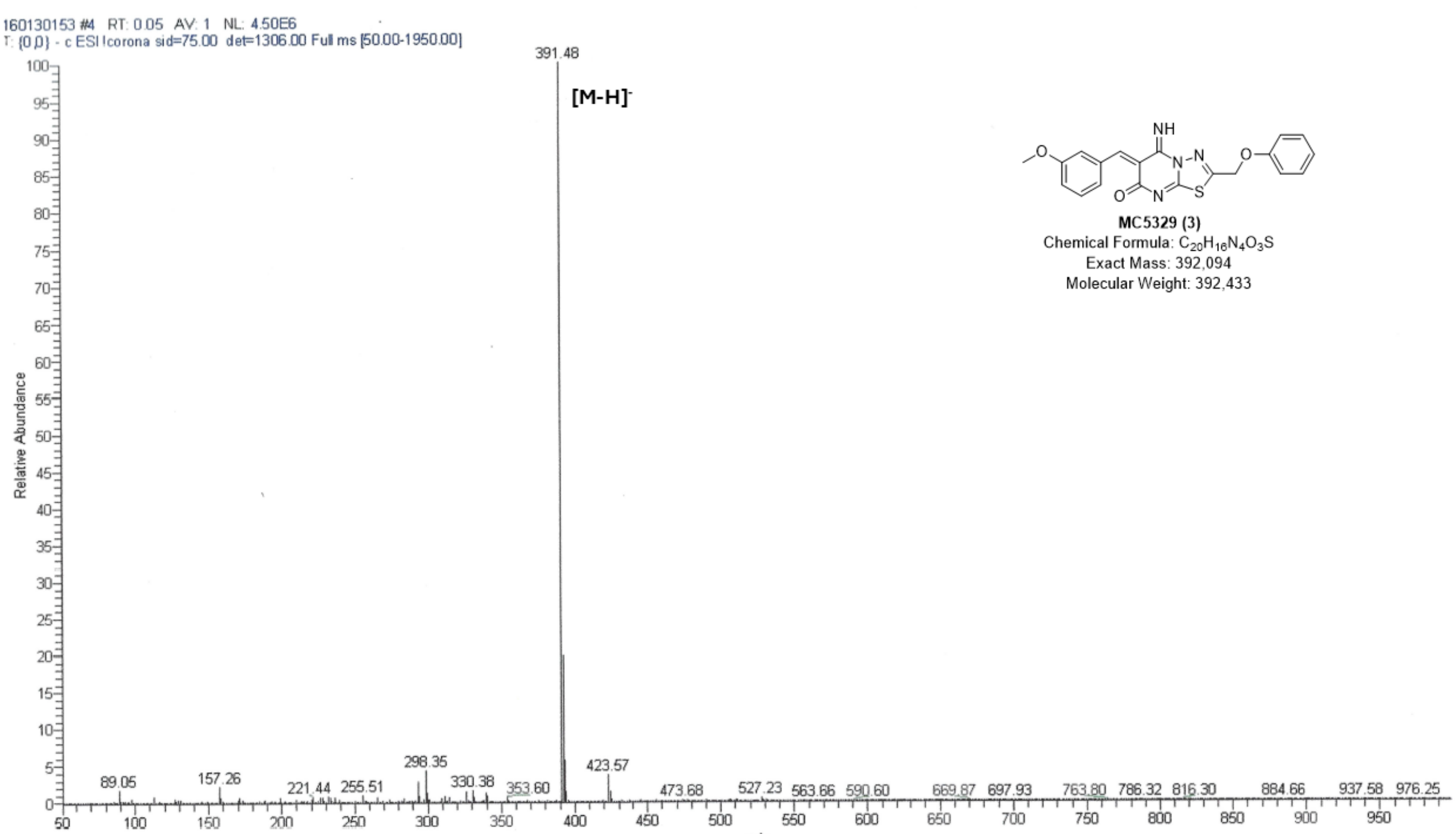
160130153 #4 RT: 0.05 AV: 1 NL: 4.50E6
T: {0,0} - c ESI !corona sid=75.00 det=1306.00 Full ms [50.00-1950.00]
391.48
[M-H]⁻
MC5329 (3)
Chemical Formula: $C_{20}H_{16}N_4O_3S$
Exact Mass: 392,094
Molecular Weight: 392,433
Relative Abundance
m/z
89.05 157.26 221.44 255.51 298.35 330.38 353.60 423.57 473.68 527.23 563.66 590.60 669.87 697.93 763.80 786.32 816.30 884.66 937.58 976.25

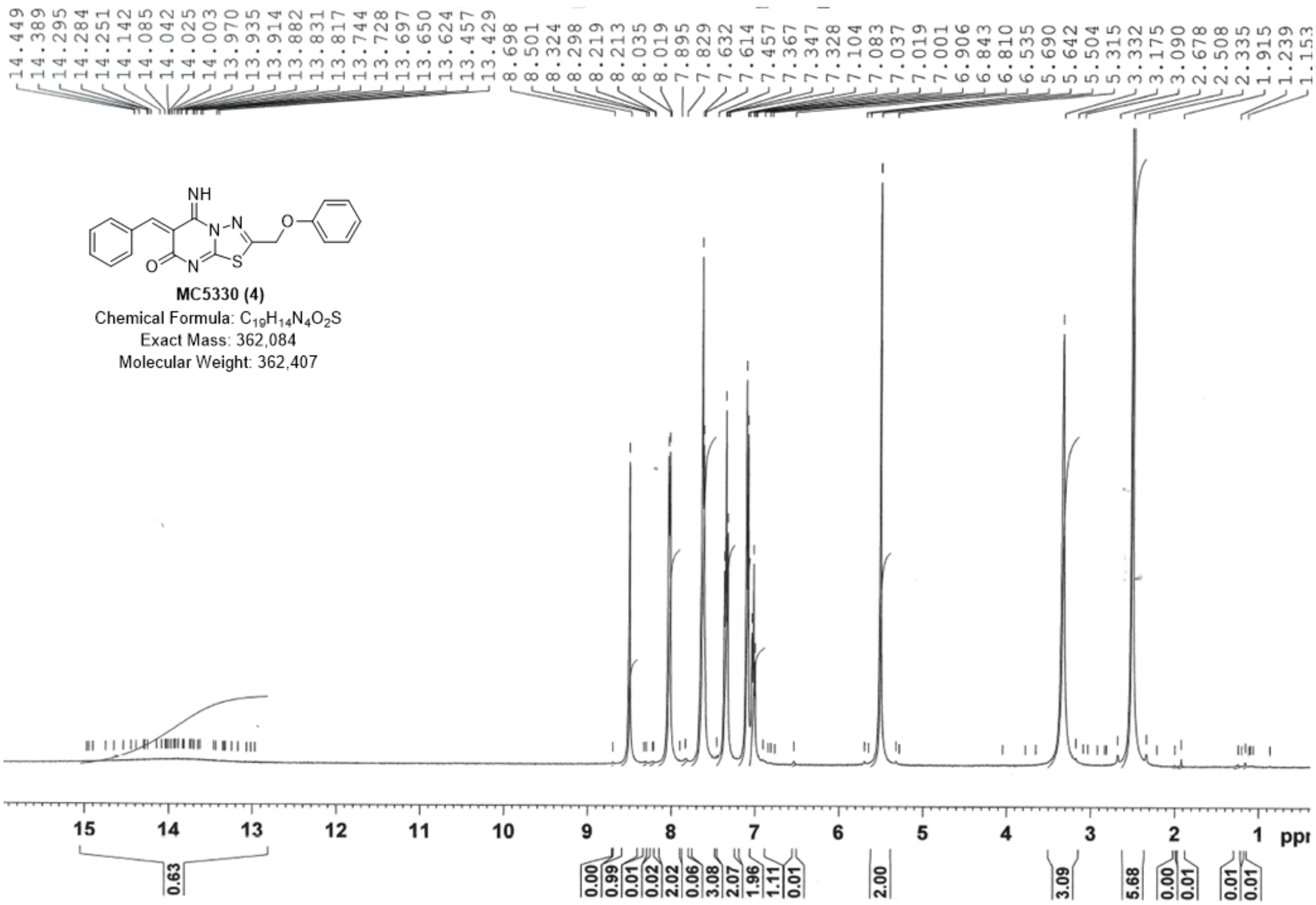
14.449 14.389 14.295 14.284 14.251 14.142 14.085 14.042 14.025 14.003 13.970 13.935 13.914 13.882 13.831 13.817 13.744 13.728 13.697 13.650 13.624 13.457 13.429 8.698 8.501 8.324 8.298 8.219 8.213 8.035 8.019 7.895 7.829 7.632 7.614 7.457 7.367 7.347 7.328 7.104 7.083 7.037 7.019 7.001 6.906 6.843 6.810 6.535 5.690 5.642 5.504 5.315 3.332 3.175 3.090 2.678 2.508 2.335 1.915 1.239 1.153
NH
O
N
N
S
MC5330 (4)
Chemical Formula: $C_{19}H_{14}N_4O_2S$
Exact Mass: 362,084
Molecular Weight: 362,407
15 14 13 12 11 10 9 8 7 6 5 4 3 2 1 ppm
0.63
0.00 0.99 0.01 0.02 2.02 0.06 3.08 2.07 1.96 1.11 0.01
2.00
3.09
5.68 0.00 0.01
0.01 0.01

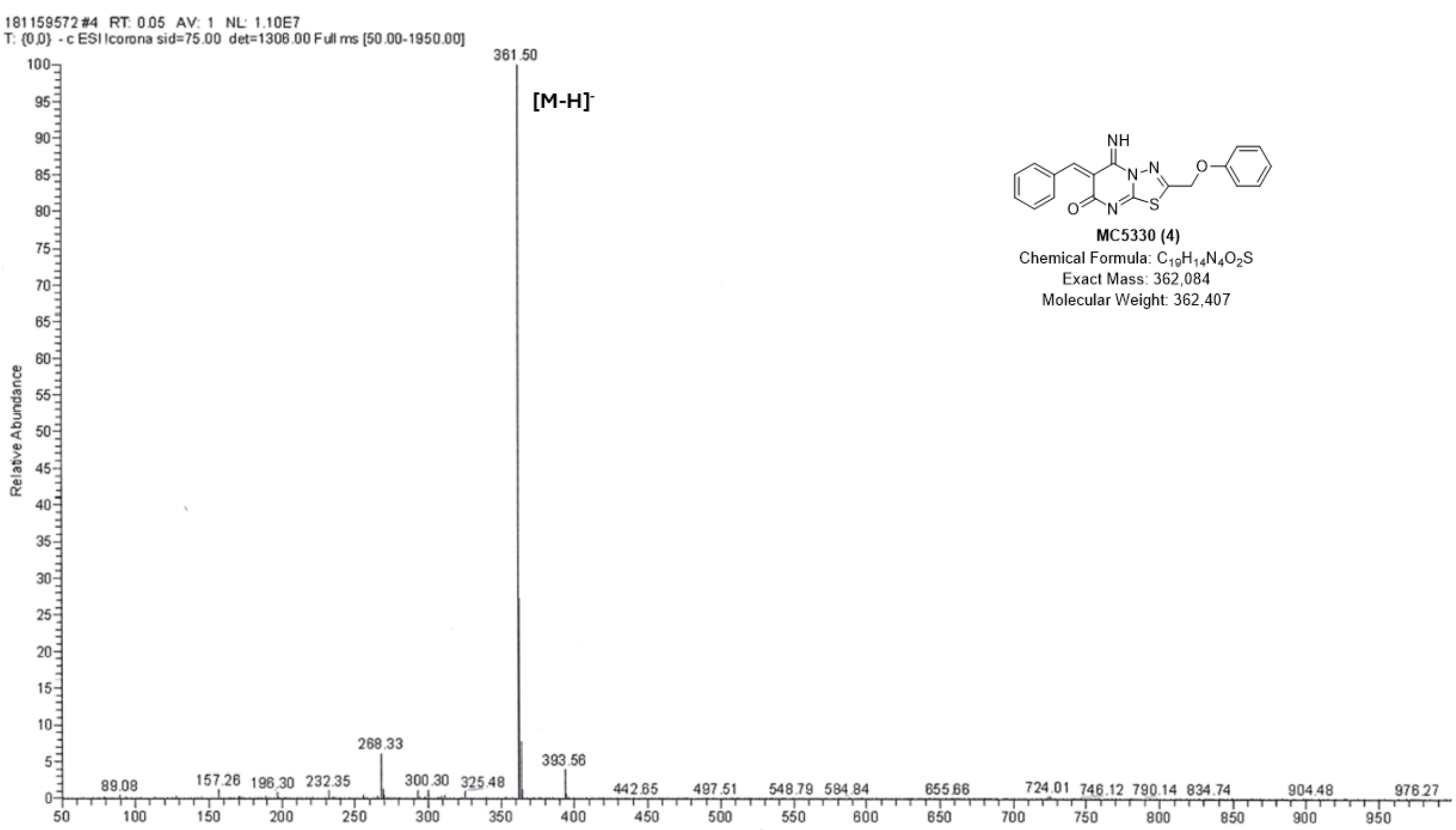
181159572 #4 RT: 0.05 AV: 1 NL: 1.10E7
T: {0,0} - c ESI !corona sid=75.00 det=1306.00 Full ms [50.00-1950.00]
361.50
[M-H]-
Relative Abundance
268.33
393.56
89.08 157.26 196.30 232.35 300.30 325.48 442.85 497.51 548.79 584.84 655.86 724.01 746.12 790.14 834.74 904.48 976.27
m/z
NH
O
N
S
MC5330 (4)
Chemical Formula: $C_{19}H_{14}N_4O_2S$
Exact Mass: 362,084
Molecular Weight: 362,407

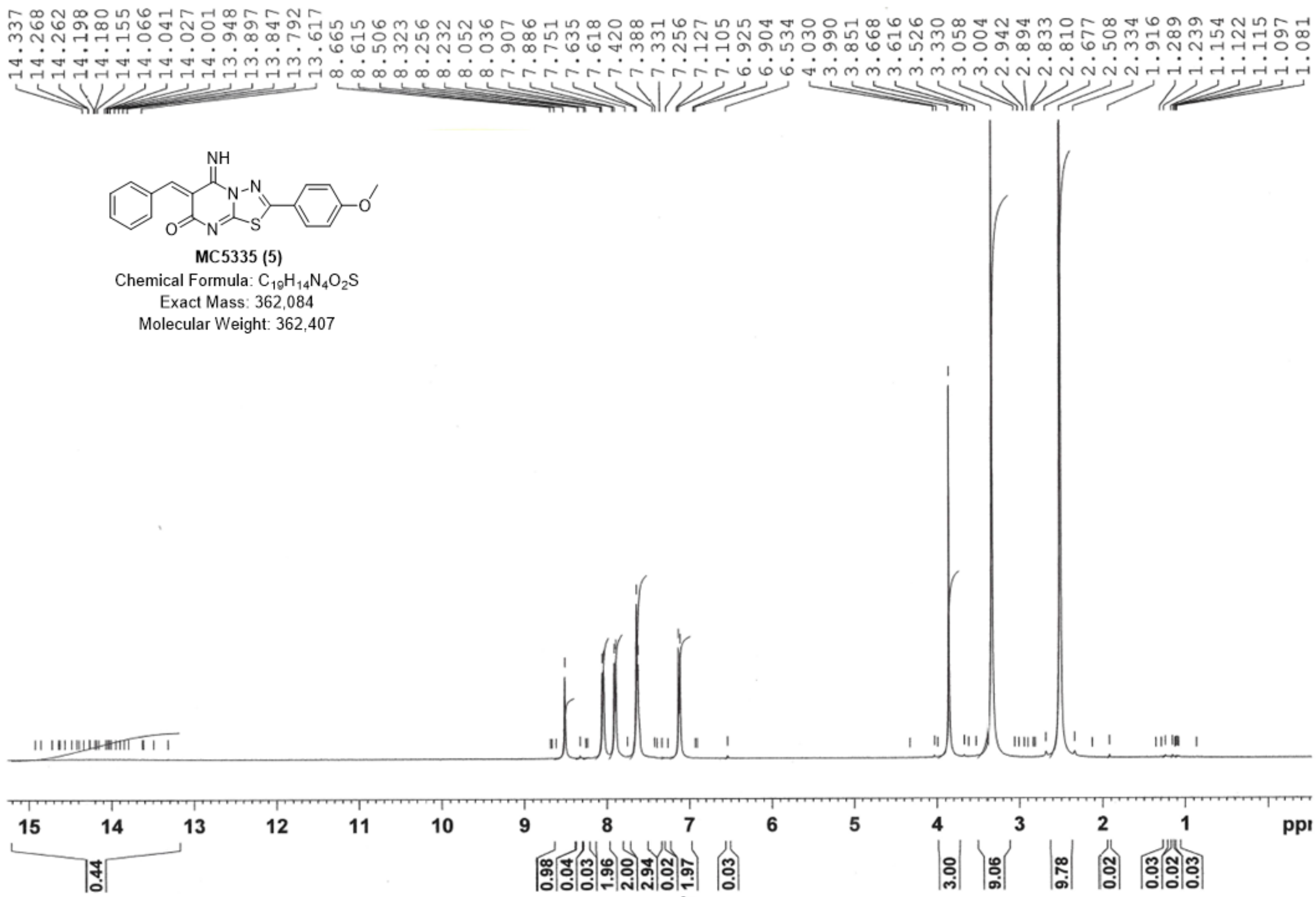
MC5335 (5)
Chemical Formula: $C_{19}H_{14}N_4O_2S$
Exact Mass: 362,084
Molecular Weight: 362,407
ppm

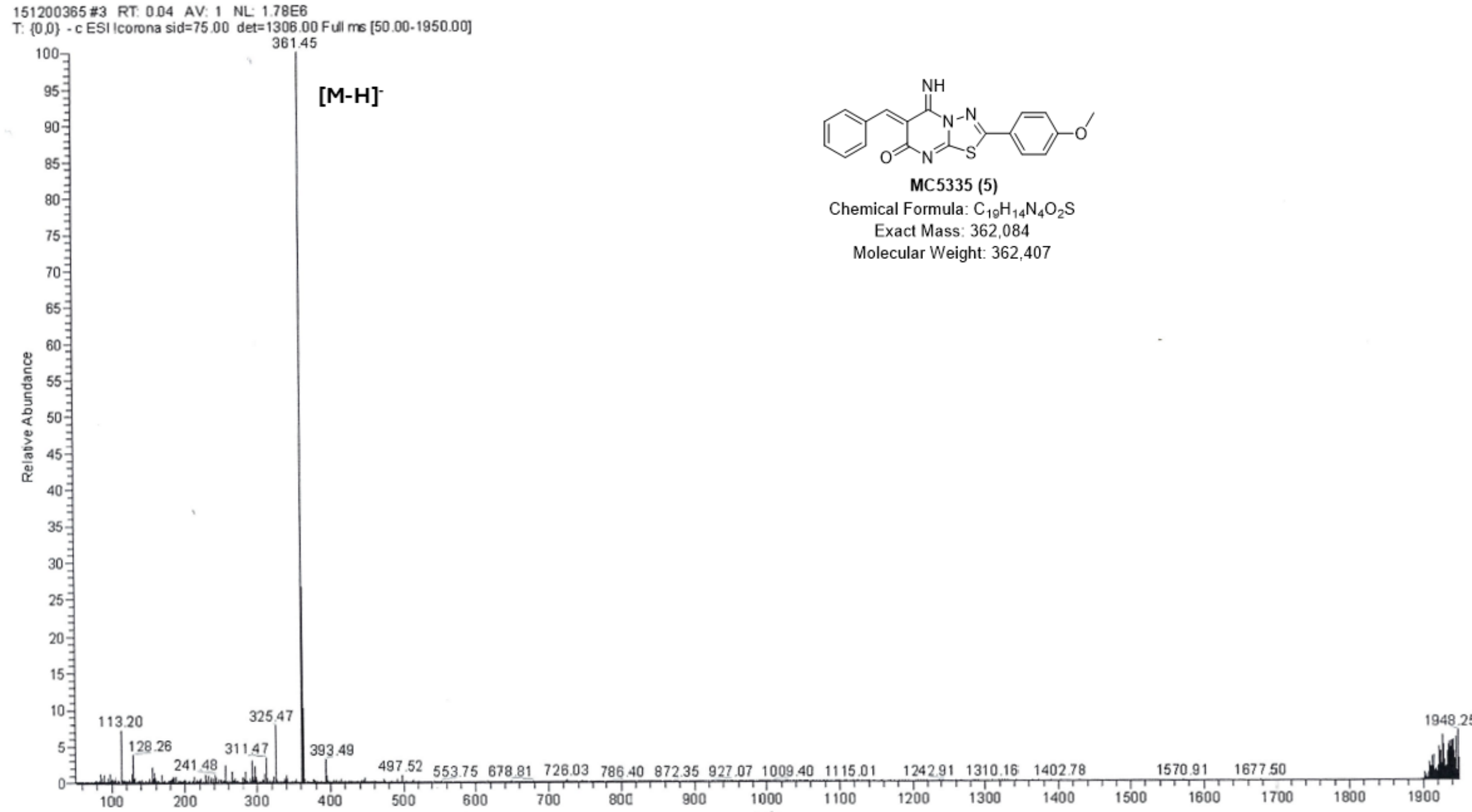
151200365 #3 RT: 0.04 AV: 1 NL: 1.78E6
T: {0,0} - c ESI !corona sid=75.00 det=1306.00 Full ms [50.00-1950.00]
[M-H]$^-$
Relative Abundance
m/z
MC5335 (5)
Chemical Formula: $C_{19}H_{14}N_4O_2S$
Exact Mass: 362,084
Molecular Weight: 362,407

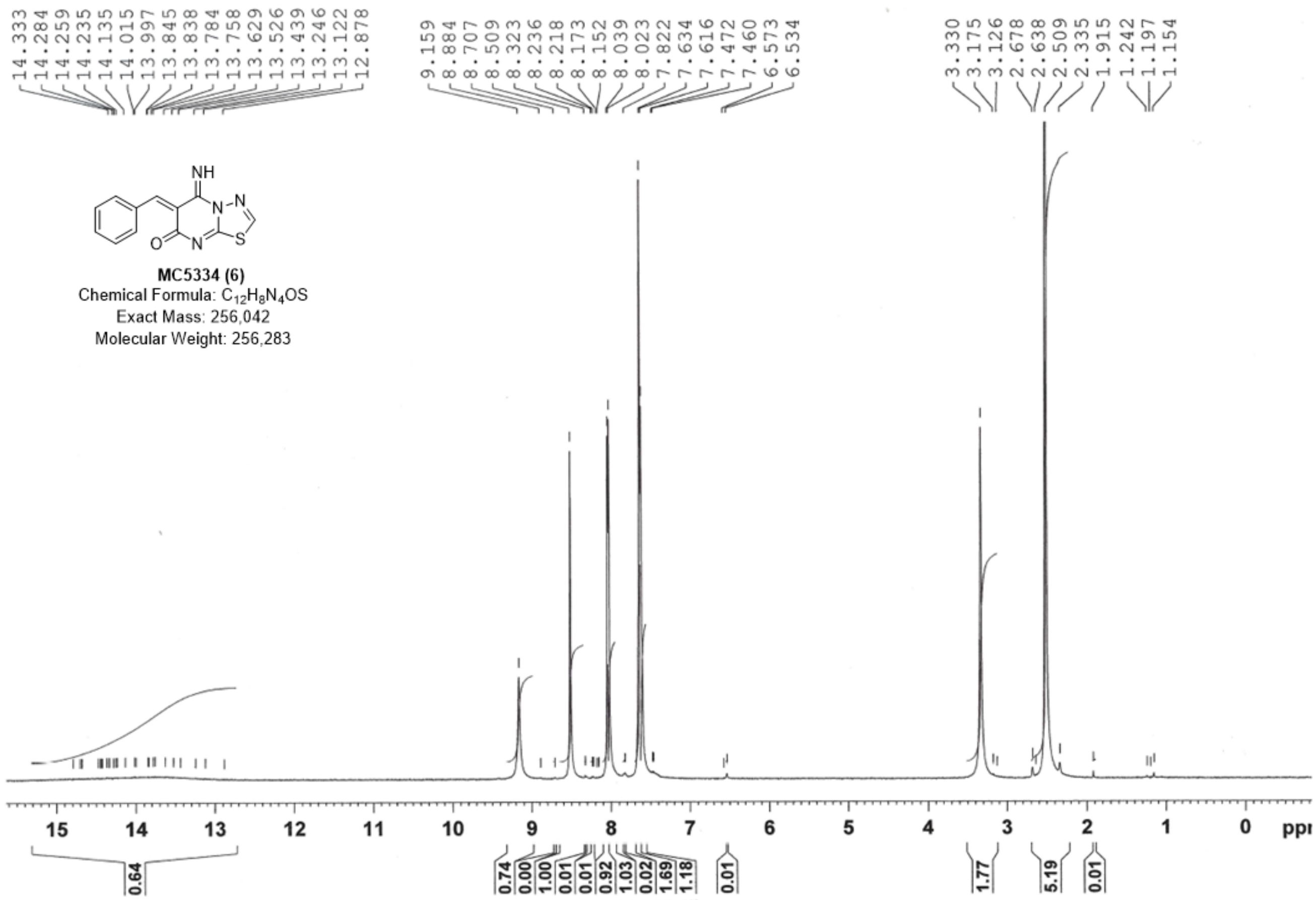

MC5334 (6)
Chemical Formula: $C_{12}H_8N_4OS$
Exact Mass: 256,042
Molecular Weight: 256,283

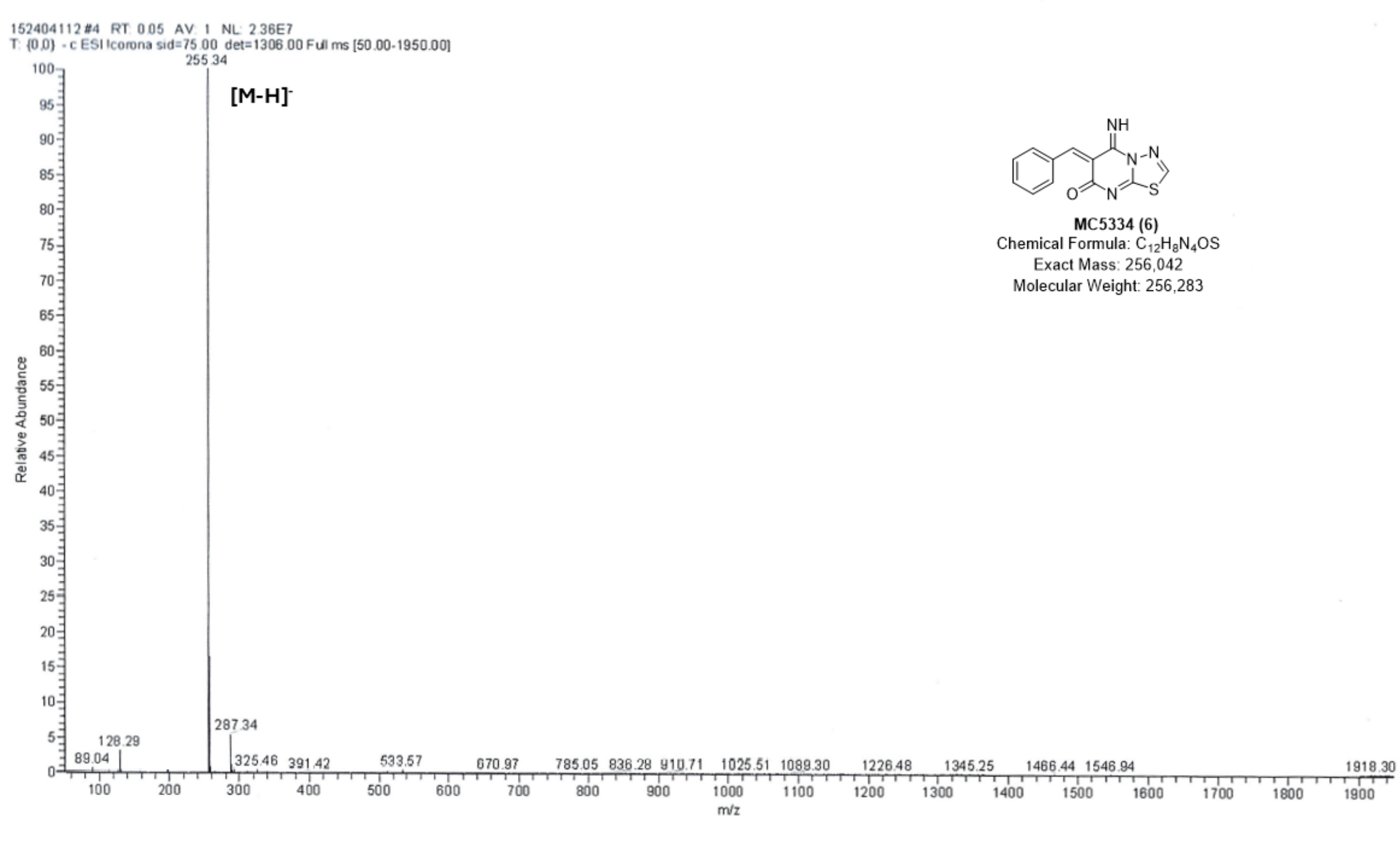

[M-H]-
255.34
287.34
128.29
Relative Abundance
m/z
MC5334 (6)
Chemical Formula: $C_{12}H_8N_4OS$
Exact Mass: 256,042
Molecular Weight: 256,283

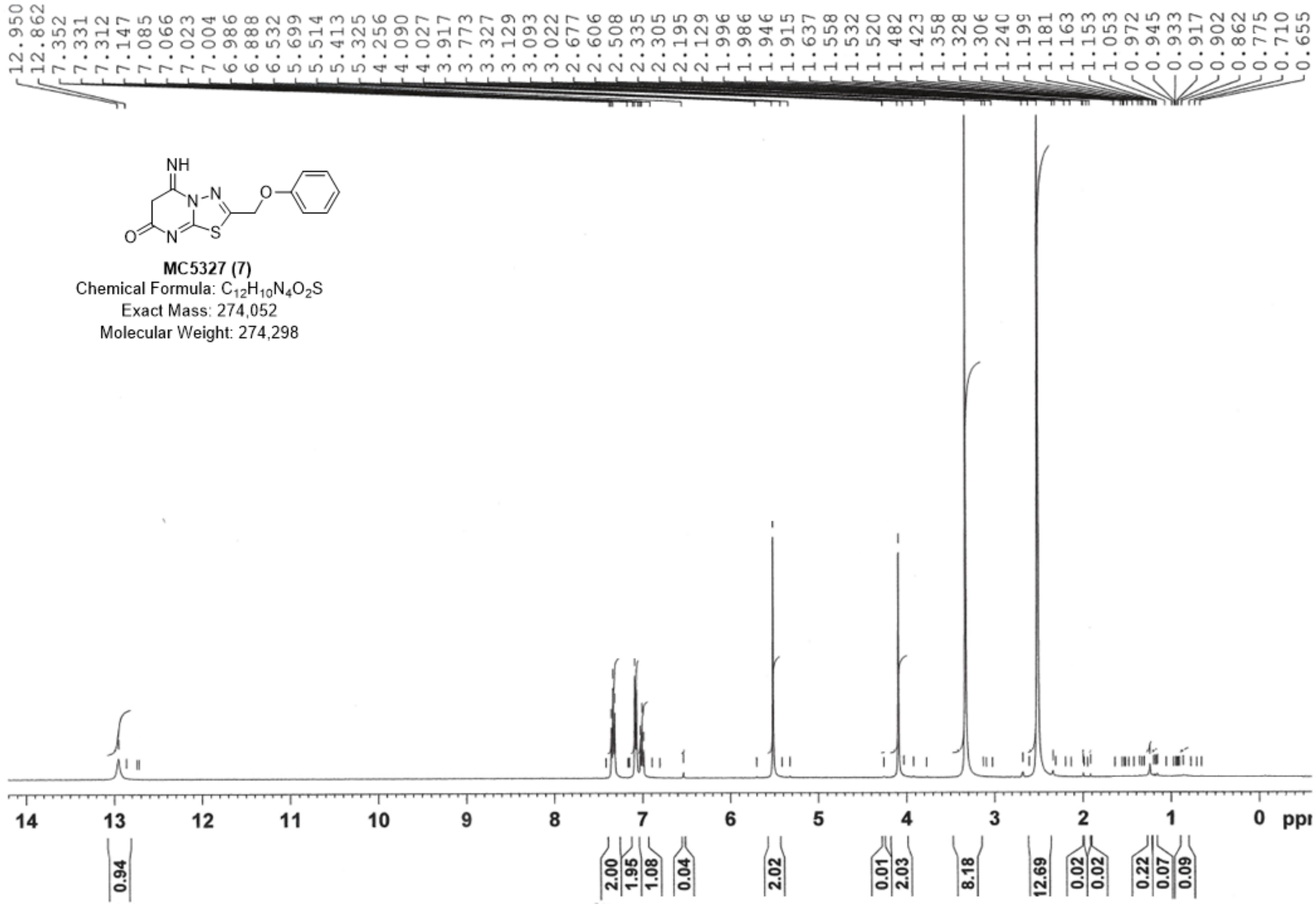
12.950
12.862
7.352
7.331
7.312
7.147
7.085
7.066
7.023
7.004
6.986
6.888
6.532
5.699
5.514
5.413
5.325
4.256
4.090
4.027
3.917
3.773
3.327
3.129
3.093
3.022
2.677
2.606
2.508
2.335
2.305
2.195
2.129
1.996
1.986
1.946
1.915
1.637
1.558
1.532
1.520
1.482
1.423
1.358
1.328
1.306
1.240
1.199
1.181
1.163
1.153
1.053
0.972
0.945
0.933
0.917
0.902
0.862
0.775
0.710
0.655
NH
O
N
N
N
S
O
MC5327 (7)
Chemical Formula: C12H10N4O2S
Exact Mass: 274,052
Molecular Weight: 274,298
14 13 12 11 10 9 8 7 6 5 4 3 2 1 0 ppm
0.94
2.00
1.95
1.08
0.04
2.02
0.01
2.03
8.18
12.69
0.02
0.02
0.22
0.07
0.09

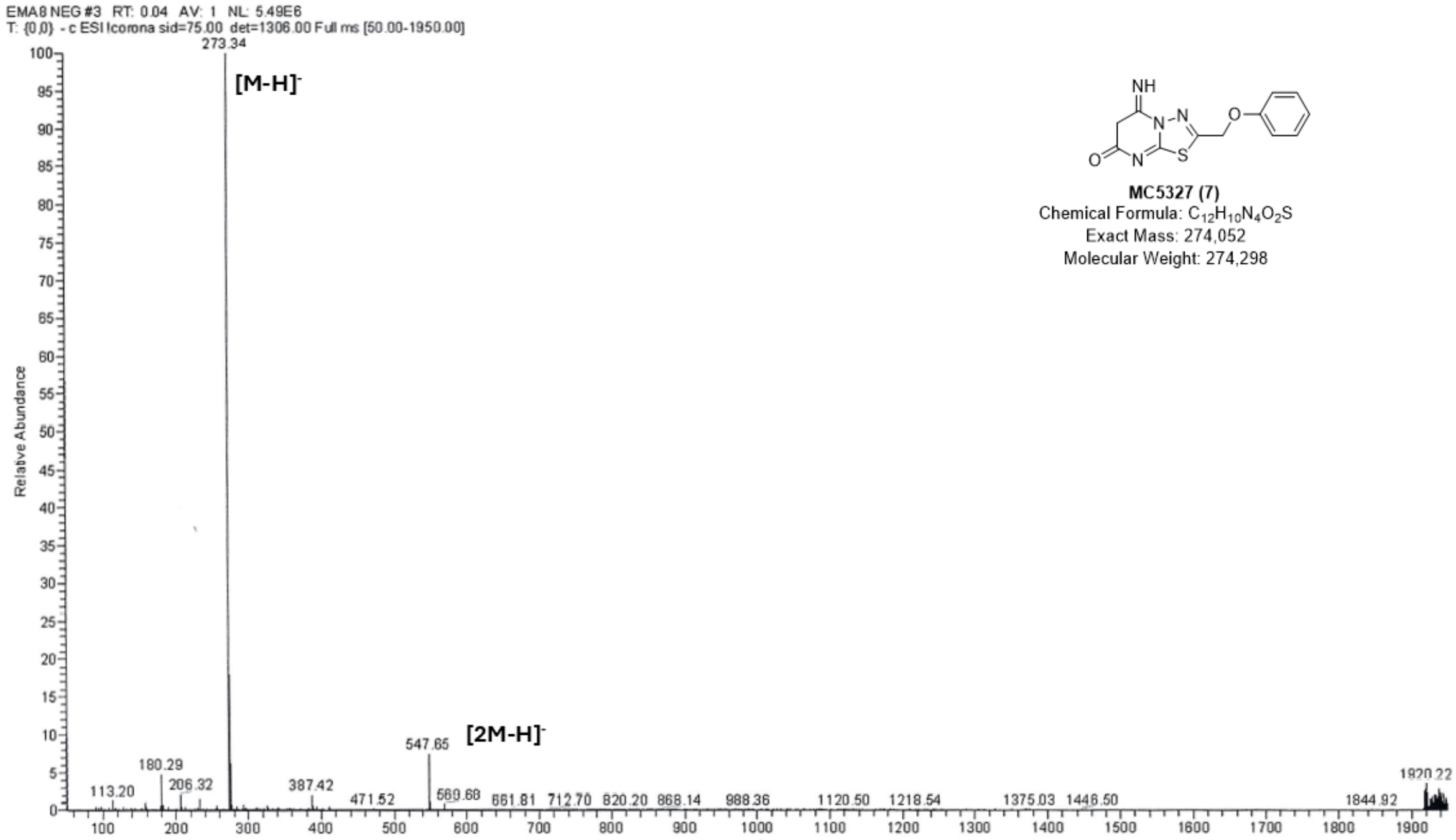
EMA8 NEG #3 RT: 0.04 AV: 1 NL: 5.49E6
T: {0,0} - c ESI Icorona sid=75.00 det=1306.00 Full ms [50.00-1950.00]
273.34
[M-H]-
547.65
[2M-H]-
113.20
180.29
206.32
387.42
471.52
569.68
661.81
712.70
820.20
868.14
988.36
1120.50
1218.54
1375.03
1446.50
1844.92
1920.22
Relative Abundance
m/z
MC5327 (7)
Chemical Formula: C12H10N4O2S
Exact Mass: 274,052
Molecular Weight: 274,298

Biochemistry

Generation of transgenic *Drosophila* expressing human NOX isozymes. Transgenic *Drosophila melanogaster* lines expressing individual human Nox isozymes were generated for inhibitor screening. To specifically inhibit Nox isozymes, transgenic *Drosophila* Duox knockdown lines were established that over-expressed human Nox1, 2, 4, 5, or Duox1. Expression of each human Nox isozyme was driven by the daughterless (Da)-GAL4 system. The presence of human Nox1, 2, 4, 5, and Duox1 isozymes in each transgenic line was validated via real-time quantitative PCR (qPCR). The genotypes used were human Nox1 (*UAS-hNox1/UAS-dDuox-RNAi; Da-GAL4/+*), human Nox2 (*UAS-hNox2/UAS-dDuox-RNAi; Da-GAL4/+*), human Nox4 (*UAS-hNox4/UAS-dDuox-RNAi; Da-GAL4/+*), human Nox5 (*UAS-hNox5/UAS-dDuox-RNAi; Da-GAL4/+*), and human Duox1 (*UAS-hDuox1/UAS-dDuox-RNAi; Da-GAL4/+*). Adult flies were homogenized in pre-chilled phosphate-buffered saline (PBS) supplemented with the protease inhibitors aprotinin and leupeptin. Following homogenization, membrane fractions were prepared, aliquoted, and preserved at −80°C until further use.

Lucigenin chemiluminescence assay for Nox inhibitor screening. For activity measurements, frozen membrane fractions were thawed and diluted in PBS immediately before the assay. Test compounds were dissolved in DMSO and serially diluted to obtain final concentrations ranging from 100 μM to 1 nM. Membrane preparations (10 μL) were mixed with compound solutions (4 μL) in white 96-well microplates and incubated for 10 min at room temperature with gentle shaking. The chemiluminescence reaction mixture consisted of 20 mM HEPES buffer (pH 7.4), NADPH (β-nicotinamide adenine dinucleotide phosphate hydrate; TGI, T0521), and lucigenin (N,N′-dimethyl-9,9′-biacridinium dinitrate; Sigma-Aldrich, M8010). After compound pre-incubation, 86 μL of the reaction mixture was added to each well, resulting in a final assay volume of 100 μL. The final concentrations of NADPH and lucigenin were adjusted to 500 μM and 400 μM, respectively. Chemiluminescence signals were monitored using a SpectraMax iD3 microplate reader (Molecular Devices, San Jose, CA, USA) at

1-min intervals for a total of 10 min. Data were recorded as relative luminescence units (RLU), and the mean value obtained from the measurement period was used for subsequent analysis. The inhibitory activity of each compound was calculated according to the following equation: Inhibition (%) = [(RLU_DMSO − RLU_Compound) / RLU_DMSO] × 100. Dose–response curves were generated by nonlinear regression analysis, and IC50 values were determined using GraphPad Prism 8 software (GraphPad Software, San Diego, CA, USA).

Computational procedures

Boltz-2 software. Protein–ligand complex structures were predicted using Boltz-2, a foundation tool that predicts biomolecular complexes via a deep learning–based co-folding framework.[1] Boltz-2 integrates protein and ligand representations within a unified architecture, enabling simultaneous inference of complex geometry and affinity, while incorporating experimental conditioning, distance constraints, and template information to guide structure prediction. For this purpose, protein amino acid sequences were retrieved from UniProt, while ligand structures were provided as SMILES strings. Specifically, the canonical human isoform sequence was selected for NOX1, while for NOX5 the β variant was chosen, as its cryo-EM structure was recently determined.[2] Where available, literature-derived and AF2BIND-based constraints were also applied to improve the reliability of the predicted binding modes. Predictions were generated with inference-time potentials (--use_potentials) enabled and structures were refined through 5 recycling iterations (--recyling_steps) to improve the physical plausibility of the predicted complexes. Evaluation of results was based on Boltz-2 protein-ligand complex confidence value, indicated with values ranging from 0 and 1.

AF2BIND. AF2bind is a logistic regression classifier trained on AlphaFold2-derived pair representations that assigns per-residue probabilities of small-molecule contact, thereby enabling the identification and clustering of putative binding-site residues based on structural features encoded in

AlphaFold2 outputs.[3] In both NOX1 and NOX5, residues predicted above the classification threshold were combined with literature-reported functional residues to constrain and prioritize binding site regions during complex modeling.

PLIP. Protein–ligand interaction profiles for the resulting complexes were analyzed using the Protein–Ligand Interaction Profiler.[4] PLIP employs a rule-based algorithm combining structural preparation, functional group assignment, and geometric criteria to automatically detect non-covalent interactions at atomic resolution, including hydrogen bonds, hydrophobic contacts, π–π stacking, π–cation interactions, salt bridges, water bridges, and halogen bonds. This enabled systematic characterization and comparison of interaction patterns across predicted complexes for downstream structure-based drug design analyses.

Neuroprotection Assays

Cell Culture and Treatment Conditions. The human neuroblastoma cell line SH-SY5Y, widely used as an in vitro model for studying neuronal function and neurodegenerative processes, was obtained from Sigma-Aldrich (ECACC). This cell line is routinely maintained under standardized culture conditions to ensure consistent growth, high viability, and reproducible experimental outcomes.

Cells are cultured in RPMI 1640 medium supplemented with 10% heat-inactivated fetal bovine serum (FBS), which provides essential growth factors and nutrients required for cell proliferation and survival. Additionally, 2 mmol/L L-glutamine is included as an important source of nitrogen and energy metabolism support, while 1% penicillin/streptomycin antibiotic solution is added to prevent bacterial contamination.

Cultures are maintained in a humidified incubator at 37 °C with 5% CO_2, conditions that closely mimic the physiological environment of human tissues and support normal cellular function. The culture

medium is replaced every 2-3 days to ensure optimal nutrient availability and to remove metabolic waste products that could affect cell growth.

For experimental assays, cells are seeded into 96-well microplates at a density of 2.5×10^4 cells per well, allowing for high-throughput analysis with minimal reagent consumption. After seeding, cells are allowed to adhere and stabilize for 24 h prior to treatment with the test compounds, ensuring uniform experimental conditions and reliable data acquisition.

Cytotoxicity Evaluation. The cytotoxic potential of the synthesized caffeine derivatives was assessed using the MTT assay (3-(4,5-dimethylthiazol-2-yl)-2,5-diphenyltetrazolium bromide), in accordance with the method originally described by Mosmann.[5] This colorimetric assay is widely used for evaluating cell metabolic activity as an indirect measure of cell viability and cytotoxicity.

Human SH-SY5Y neuroblastoma cells were treated with a range of concentrations of the tested compounds (1, 10, 50, 100, 200 and 500 μM) and incubated for 24 h to allow potential cytotoxic effects to develop. Following exposure, 100 μL of MTT solution (5 mg/mL prepared in culture medium) was added to each well. The plates were then incubated for an additional 3 h at 37 °C, during which metabolically active cells reduced the MTT reagent to insoluble purple formazan crystals.

After incubation, the formed formazan crystals were dissolved by adding 100 μL of dimethyl sulfoxide (DMSO) to each well, ensuring complete solubilization and uniform color development. The optical density was subsequently measured at 570 nm, with a reference/background wavelength of 690 nm, using a Synergy 2 microplate reader (BioTek Instruments, Inc., Winooski, VT, USA).

All experimental conditions were performed in six independent replicates ($n = 6$) to ensure statistical reliability. Cell viability was calculated and expressed as a percentage relative to untreated control cells, which were considered as 100% viability.

Hydrogen Peroxide-Induced Oxidative Stress Model. An in vitro oxidative stress model was established using human SH-SY5Y neuroblastoma cells to evaluate the potential neuroprotective and

antioxidant effects of the tested compounds under conditions mimicking neuronal oxidative injury associated with neurodegenerative disorders. SH-SY5Y cells were seeded into 96-well plates at a density of 3.5×10^4 cells/well and incubated for 24 h at 37 °C in a humidified atmosphere containing 5% CO_2. Cells were subsequently pretreated with the tested compounds at concentrations of 1, 5, and 10 μM for 90 min prior to oxidative stress induction. Oxidative damage was induced using freshly prepared hydrogen peroxide (H_2O_2) at a final concentration of 1 mM for 15 min under standard culture conditions. Following treatment, the medium was replaced with fresh culture medium, and cells were further incubated for 24 h. Cell viability was assessed using the MTT assay, based on the metabolic reduction of MTT to insoluble formazan crystals by viable cells. Protective effects were expressed as percentage protection relative to two reference groups: cells treated only with H_2O_2 were considered as 0% protection, while untreated control cells represented 100% viability. All experimental conditions were performed in six independent replicates (n = 6) to ensure reproducibility and statistical reliability of the results.

L-Glutamate-Induced Neurotoxicity Model. The neuroprotective potential of the compounds was further assessed using an L-glutamate-induced excitotoxicity model. SH-SY5Y cells were seeded at 3.5×10^4 cells/well in 96-well plates and incubated for 24 h under standard conditions. After medium removal, cells were pretreated with test compounds at concentrations of 1, 5, and 10 μM for 30 min. Treatment solutions were then discarded, and cells were washed with PBS before exposure to 65.0 mM L-glutamate for 24 h to induce neurotoxicity. The L-glutamate stock solution (65.0 mM) was prepared by initial dissolution of L-glutamate in hydrochloric acid, followed by careful adjustment of the pH to 7.4 using sodium hydroxide under continuous monitoring with a calibrated pH meter. The final concentration was achieved by adjusting the volume with phosphate-buffered saline (PBS).

Cell viability was assessed by the MTT assay. All treatments were performed in six replicates (n = 6). Control groups included untreated cells (intact control) and cells treated with L-glutamate alone (damaged control), which served as reference points for evaluating neuroprotection.

MPP^+-Induced Model of Neurotoxicity. Cells were seeded in 96-well plates at a density of 1.5×10^4 cells per well and allowed to adhere overnight. After 24 h, the experimental treatment was initiated. Cells were exposed to 1.5 mM MPP^+ for 24 h in the presence or absence of the tested compounds, applied at concentrations of 1, 5, and 10 μM. The MPP^+-treated group was used as a toxic control, as this concentration reduced cell viability to approximately 65%, providing a consistent model of dopaminergic neurotoxicity for evaluation of potential protective effects. The MPP^+ stock solution (10 mM) was freshly prepared prior to each experiment by dissolving the compound in cell culture medium.

Animals. 4 male Wistar rats with body weight 200-250 g were used. They were housed in plexiglass cages (3 per cage) in a 12/12 light/dark cycle under standard laboratory conditions (ambient temperature 20 °C ± 2 °C and humidity 72% ± 4%) with free access to water and standard pelleted rat food 53-3, produced according to ISO 9001:2008. The animals were purchased from the National Breeding Centre, Sofia, Bulgaria. Seven days' acclimatization was allowed before the commencement of the study, and a veterinary physician monitored the health of the animals regularly. Vivarium (certificate of registration of farm № 0072/01.08.2007) was inspected by the Bulgarian Drug Agency to check the husbandry conditions (№ A-11-1081/03.11.2011). All performed procedures were approved by the Institutional Animal Care Committee of Medical University of Sofia (Bulgarian Agency for Food Safety with Permission № 190, approved on February 6, 2020, and valid till December 2026). The principles stated in the European Convention for the Protection of Vertebrate Animals used for Experimental and other Scientific Purposes (ETS 123) were strictly followed throughout the experiment. Brains were taken from rats immediately after decapitation and stored on ice until needed.

Sub-cellular in vitro studies. Two types of buffers were used for the isolation of rat brain synaptosomes and mitochondria. Buffer A was 5 mM HEPES and 0.32 M Sucrose; Buffer B was 290 mM NaCl, 0.95 mM $MgCl_2 \times 2 H_2O$, 10 mM KCl, 2.4 mM $CaCl_2 \times 2 H_2O$, 2.1 mM NaH_2PO_4, 44 mM HEPES, 13 mM D-Glucose. For the gradient centrifugation, Percoll reagent was used. A stock solution of 90% Percoll was used to prepare 16%, 10% and 7.5% solutions. All solutions were used as described below. Immediately after harvesting, synaptosomes, microsomes and mitochondria were incubated with the exam compounds. The protein measurement of all sub-cellular fractions was made by method of Lowry et al., 1951.[6]

Rat brain synaptosomes – isolation and incubation. The synaptosomes were prepared from rat brains by multiple subcellular fractionations using a Percoll gradient.[7]

Model of 6-OHDA-induced neurotoxicity. This in vitro model resembles the neurodegenerative processes occurring in Parkinson's disease. Dopamine metabolism and oxidation lead to the formation of reactive oxygen species (ROS) and reactive quinones. These processes were catalyzed by MAO-B enzyme. They induce dopamine neurotoxicity and neurodegeneration.[8] The synaptosomes were incubated with 150 μM 6-OHDA for 1 h.

Synaptosomal viability. After the incubation, the synaptosomes were centrifuged three times at 15 000×g for 1 min. MTT-test was performed to determine synaptosomal vitality by method described by Mungarro-Menchaca et al. (2002).[9]

Determination of reduced glutathione (GSH). The level of GSH was determined by measuring the non-protein SH-groups after precipitation of the proteins with trichloroacetic acid (TCA). The presence of thiols in the supernatant was determined using Elmman reagent. The resulting yellow colour was measured spectrophotometrically ($\lambda = 412$ nm).[10]

Rat brain microsomes – preparation. The brain was homogenized in 9 vol. of 0.1 M Tris buffer, containing: 0.1 mM Dithiothreitol, 0.1 mM Phenylmethylsulfonyl fluoride, 0.2 mM EDTA, 1.15% KCl

and 20% (v/v) glycerol at pH 7.4. The homogenate was centrifuged twice at 17 000×g for 30 min. The supernatants from both centrifugations were combined and centrifuged twice at 100 000×g for 1 h. The resulting pellet was frozen in the Tris until needed.[11]

$FeSO_4$/ascorbic acid-induced lipid peroxidation (LPO). The LPO was started with a solution of 20 µM iron sulphate and 500 µM ascorbic acid for 1 h.[12]

Malondialdehyde (MDA) assay. The quantity of the lipid peroxidation product MDA was assessed, using the method, described by Deby and Goutier (1990).[13]

Rat brain mitochondria – isolation. The mitochondria were prepared by multiple, differential fractionation using a Percoll gradient.[14]

***tert*-Butyl hydroperoxide (*t*-BuOOH)-induced oxidative stress.** The mitochondria were incubated with 75 µM *t*-BuOOH for 1 h.[15]

Lipid peroxidation assay. After incubation of the mitochondria, 0.3 mL 0.2% TBA and 0.25 mL sulphuric acid (0.05 M) were added to stop the reaction. The tubes were maintained at 100 °C for 30 min. In the next stage, centrifugation of the tubes was carried out at 3500×g for 10 min. Assessment of the total quantity of MDA formed in each sample was measured in the supernatant at 532 nm.[16]

Measurement of GSH content. After incubation of mitochondria, 0.04% DTNB was mixed with the mitochondrial suspensions in 0.1 M of phosphate buffers (pH 7.4). The absorbance of the yellow product was measured at 412 nm.[16]

Statistical Analysis. Statistical analyses were performed using MedCalc and GraphPad Prism. Data are presented as mean ± SEM (brain microsomes, synaptosomes, and mitochondria; n = 6) or mean ± SD (cellular MTT assay; n = 6; antioxidant assays; n = 3). Non-parametric comparisons for brain tissue experiments were performed using the Mann–Whitney test in MedCalc. Cellular and chemical assay data were analyzed by one-way ANOVA followed by Dunnett's post hoc test in GraphPad Prism. Statistical significance was set at $p \leq 0.05$.

Antioxidant/ROS Scavenging Activity

Deoxyribose degradation assay. The test was performed using the method adapted from Sadowska-Bartoz et al..[17, 18] To assess oxidative interactions, we monitored the generation of thiobarbituric acid-reactive substances (TBARS). The method is based on the reaction between the secondary MDA-like products and two molecules of thiobarbituric acid (TBA). The formed, pink-colored complex was subsequently spectrophotometrically determined at 532 nm. Two types of samples were prepared: control samples and test samples containing the compounds under investigation. 2-Deoxyribose (0.5 mM) was dissolved in PBS, and oxidative damage was initiated with $FeCl_2$ (0.1 mM). The mixtures were incubated at 37 ºC for 30 min. Then the reaction was stopped by adding 0.5 mL of 2.8% trichloroacetic acid and 0.5 mL of thiobarbituric acid. A second incubation was performed at 100 °C. After cooling, absorbance was measured at 532 nm, and the results were expressed as a percentage of the control samples.

Enhanced chemiluminescence for ROS scavenging activity. We used enhanced chemiluminescence methods to determine the radical-scavenging activity against biologically important reactive oxygen species (ROS). These methods are well known for their sensitivity and remarkable detection limits.[19, 20] Luminol was chosen for the assays due to its broad reactivity toward different types of ROS.[21] An LKB 1251 chemiluminometer connected to an IBM-compatible computer was used for these experiments. Scavenging activity was evaluated from the area under the chemiluminescent curve and expressed as chemiluminescent scavenging index (CL-SI, %), calculated relative to the control sample. **Superoxide anion radical scavenging activity assay**. 1 mL PB (50 mM K_2HPO_4/KH_2PO_4, pH 7.4) containing 0.1 mM luminol and the tested derivatives were prepared. Control samples were prepared without adding the tested compounds. Chemiluminescence was measured immediately after the addition of 20 µL KO_2 in DMSO for 1 min at 50 ms intervals. **Hypochlorite scavenging activity assay**. Samples

(1 mL) consisting of PB (pH 7.4), 0.1 mM luminol, 0.06 mM NaClO, and the investigated compounds were used, with buffer in control. CL emission was monitored for 1 min every 50 ms.

Spectrophotometric determination of the hydrogen peroxide scavenging activity. An ABTS/HRP colorimetric assay has been applied. It is based on the horseradish peroxidase (HRP)-catalyzed oxidation of 2,2'-azinobis-(3-ethylbenzothiazoline-6-sulfonic acid) (ABTS) in the presence of H_2O_2.[22] The presence of hydrogen peroxide scavenging compounds, results in a decrease in the extent of chromophore formation and in absorbance values relative to the controls, in which maximal radical generation is expected. The working solution comprised 2.0 mM ABTS and 0.86 nM HRP enzyme, all dissolved in 50 mM PB, pH 7.4. Absorbance was measured at 734 nm using a Genesys 50 spectrophotometer (Thermo Scientific, Madison, WI, USA). Hydrogen peroxide (1 mM) was preincubated with the tested derivatives for 15 min in the dark prior to analysis. Additionally, the scavenging activity of both compounds against $ABTS^{\bullet +}$ was also determined in the same concentration range to exclude interference (λ = 734 nm).

Anticancer Assays

Compound treatment. Compound treatment of cells started one day after seeding with a final DMSO concentration of 0.1% and was performed by nanodrop-dispensing using a Tecan Dispenser (D300e). Solvent only (0.1% DMSO) served as high control (100% viability) and staurosporine (10 μM) as low control (0% viability). Compounds were tested in singlet 10-dose mode with 3-fold serial dilution starting from a 200 μM solution, for 72 h of treatment.

Cell Viability assay. Cells were taken from the Reaction Biology Europe GmbH collection and were cultured in different media (DMEM plus 10% FCS for Caco-2 and HT-1080, RPMI plus 10% FCS for PSN1 and SU-DHL-6, and RPMI-1640 plus 10% FCS for RPMI 8226). For the assays, cells were seeded in white cell culture-treated flat and clear bottom 384-well plates and incubated at 37 °C overnight

before compounds were added. After incubation for 72 h at 37 °C at 5% CO_2, cell plates were equilibrated to room temperature for one h, CellTiter-Glo® reagent (Promega) was added, and luminescence was measured approximately one hour later using a luminometer (EnVision, PerkinElmer).

Evaluation of raw data. Raw data were converted into percent cell viability relative to the high and low control, which were set to 100% and 0%, respectively. Absolute IC_{50} values were calculated by GraphPad Prism software with a variable slope sigmoidal response fitting model using 0% viability as bottom constraint and 100% viability as top constraint. Relative IC_{50} values (with no bottom constraint) were determined if the maximum effect on many cell lines leveled out at only partially reduced viability.

Figure S1. Inhibition curves for the NOX inhibitors 1, 2, and 5

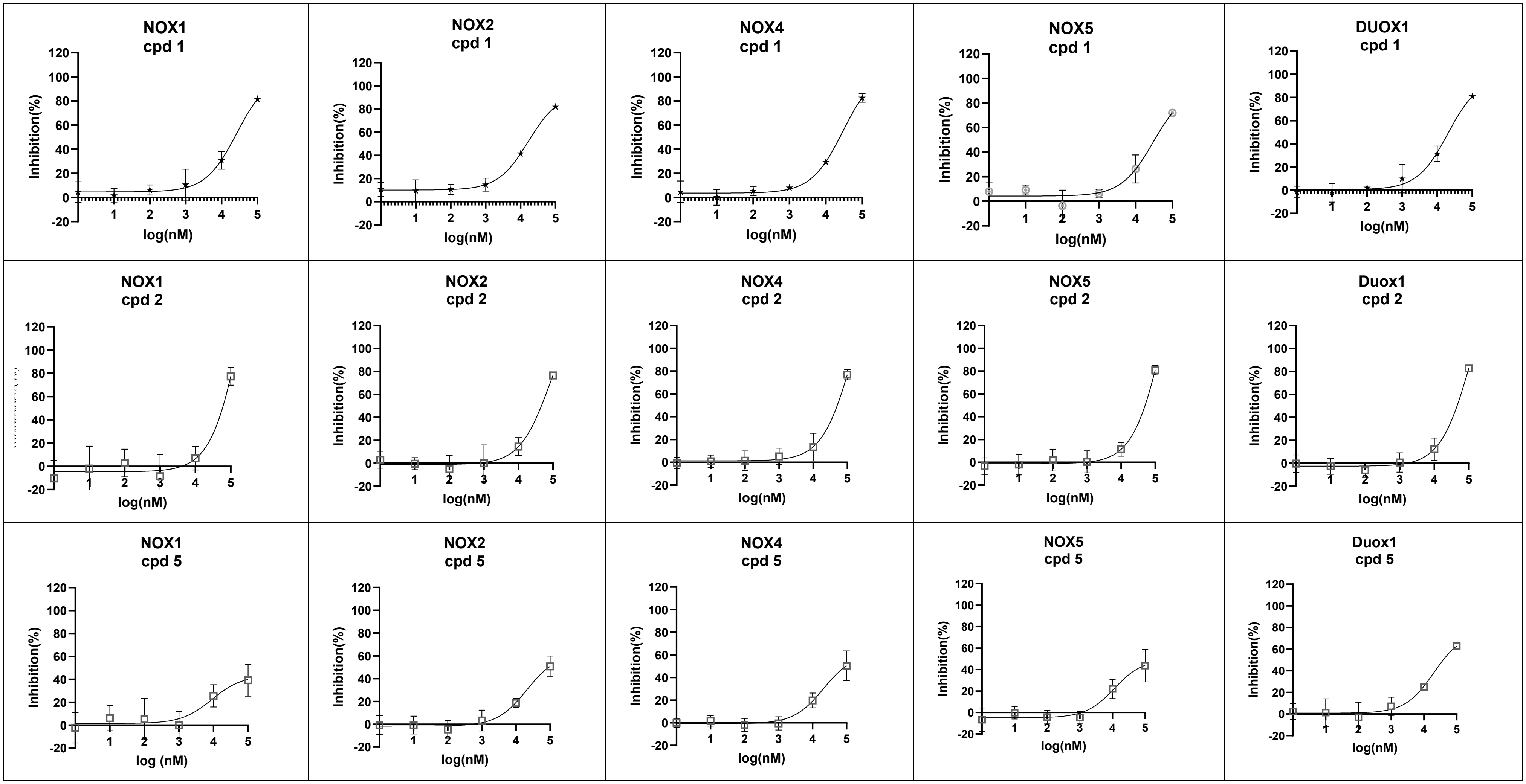

Figure S2. Stability Assays: HPLC traces of compound 1 (M11) in PBS (pH 7.4) after 0, 4, 24, and 48 h at 37 °C. Compound **1** (M11) was diluted to 50 μM in PBS from a 40× DMSO stock, incubated at 37 °C, and analyzed by analytical HPLC (detection at 280 nm; t_R = 19.5 min) under the conditions described below. Purity was 98.4% at 0 h and did not decrease significantly over the time course, indicating good stability under these conditions.

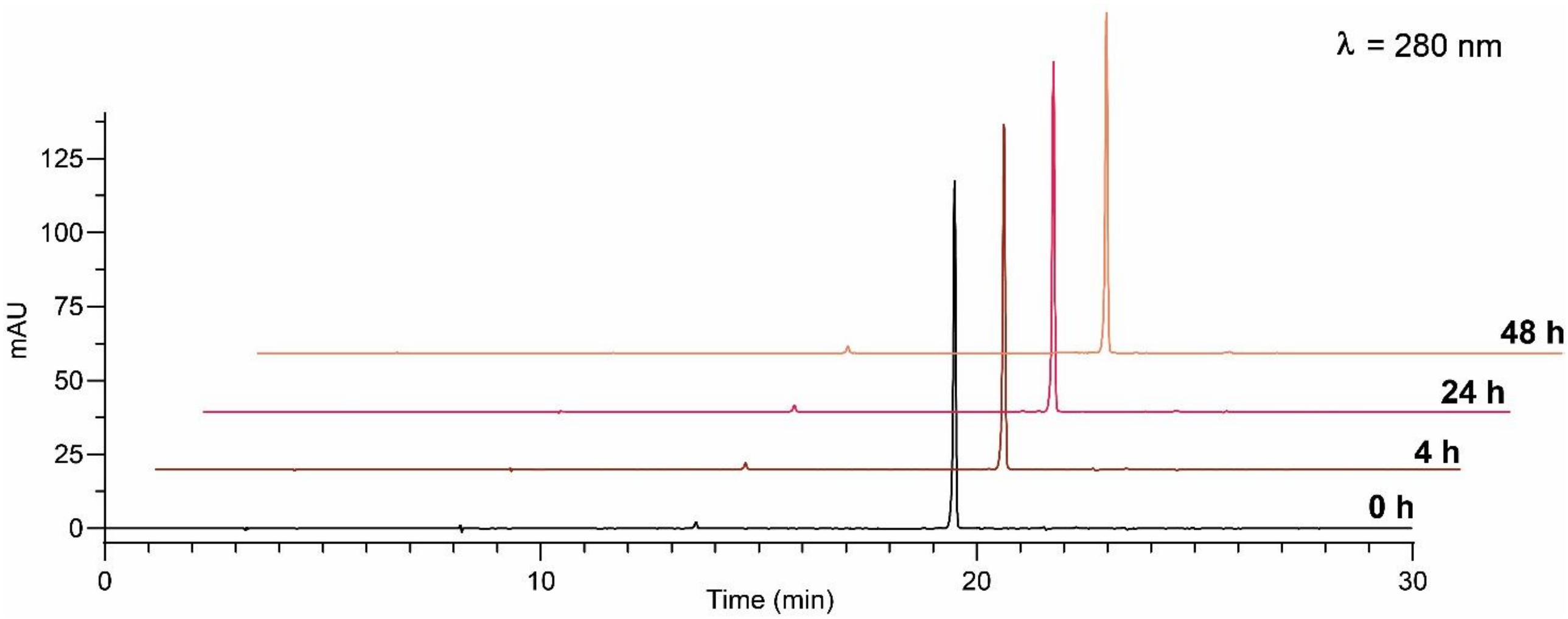

HPLC was used to determine the purity and stability of **1** following incubation in PBS for 0, 4, 24, and 48 h at 37 °C. The HPLC system consisted of a Dionex UltiMate 3000 UHPLC (Thermo Fisher) system equipped with an automatic injector, column heater and coupled with a Diode Array Detector DAD-3000 (Thermo Fisher). The analytical controls were performed on a Hypersil GOLD C18 Selectivity 5 µm (4.6 × 250 mm) HPLC Column (Thermo Fisher) in gradient elution. Eluents: (A) H_2O + 25 mM ammonium acetate (pH 8.8); (B) CH_3CN. The chromatographic run comprised 3 min at 100% solvent A/0% solvent B, a subsequent 22 min linear gradient from 0 to 100% solvent B, and a final 5 min isocratic step at 90% solvent B. The flow rate was 1.0 mL/min, and the column was kept at a constant temperature of 30 °C. Samples were dissolved in DMSO (20 mM) and then diluted in PBS at a final concentration of 50 μM and the injection volume was 20 μL.

Figure S3. ***In vitro*** **cytotoxicity evaluation of compound 1 (MC5328) and compound 5 (MC5335) on SH-SY5Y cells**

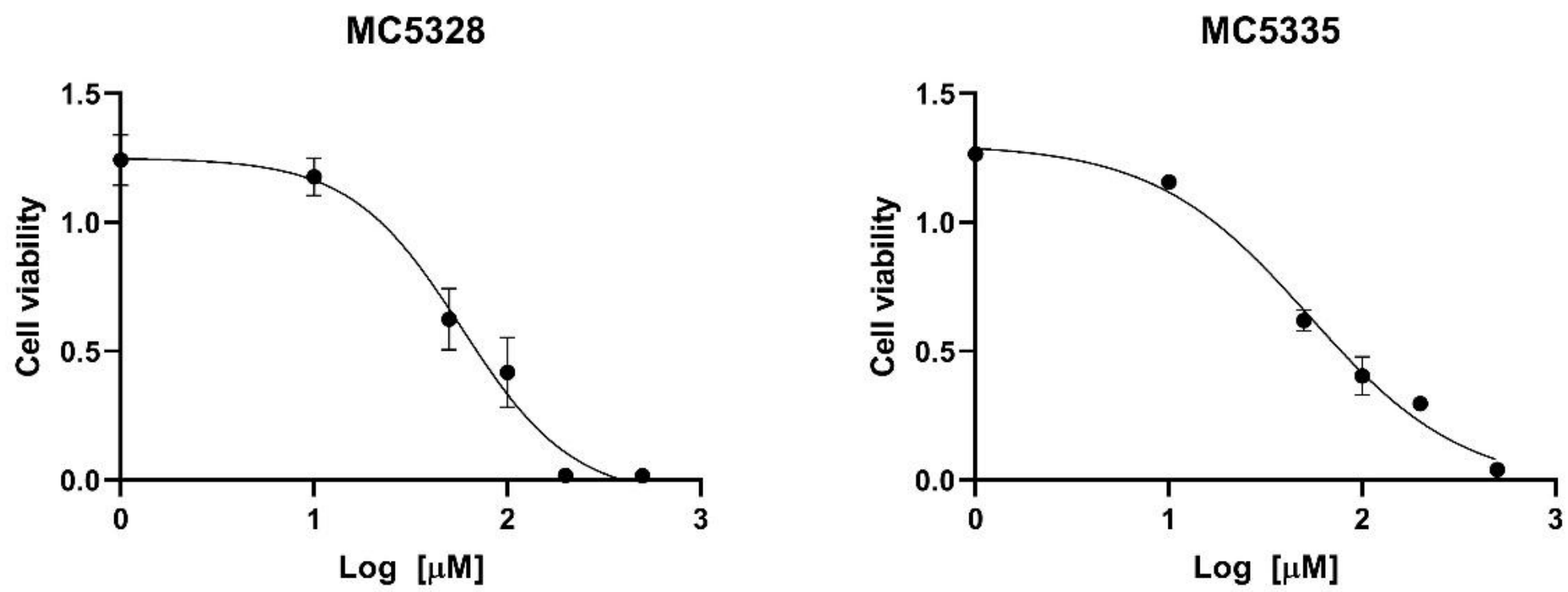

Figure S4. Antiproliferative curves for compounds 1 and 5

1	5

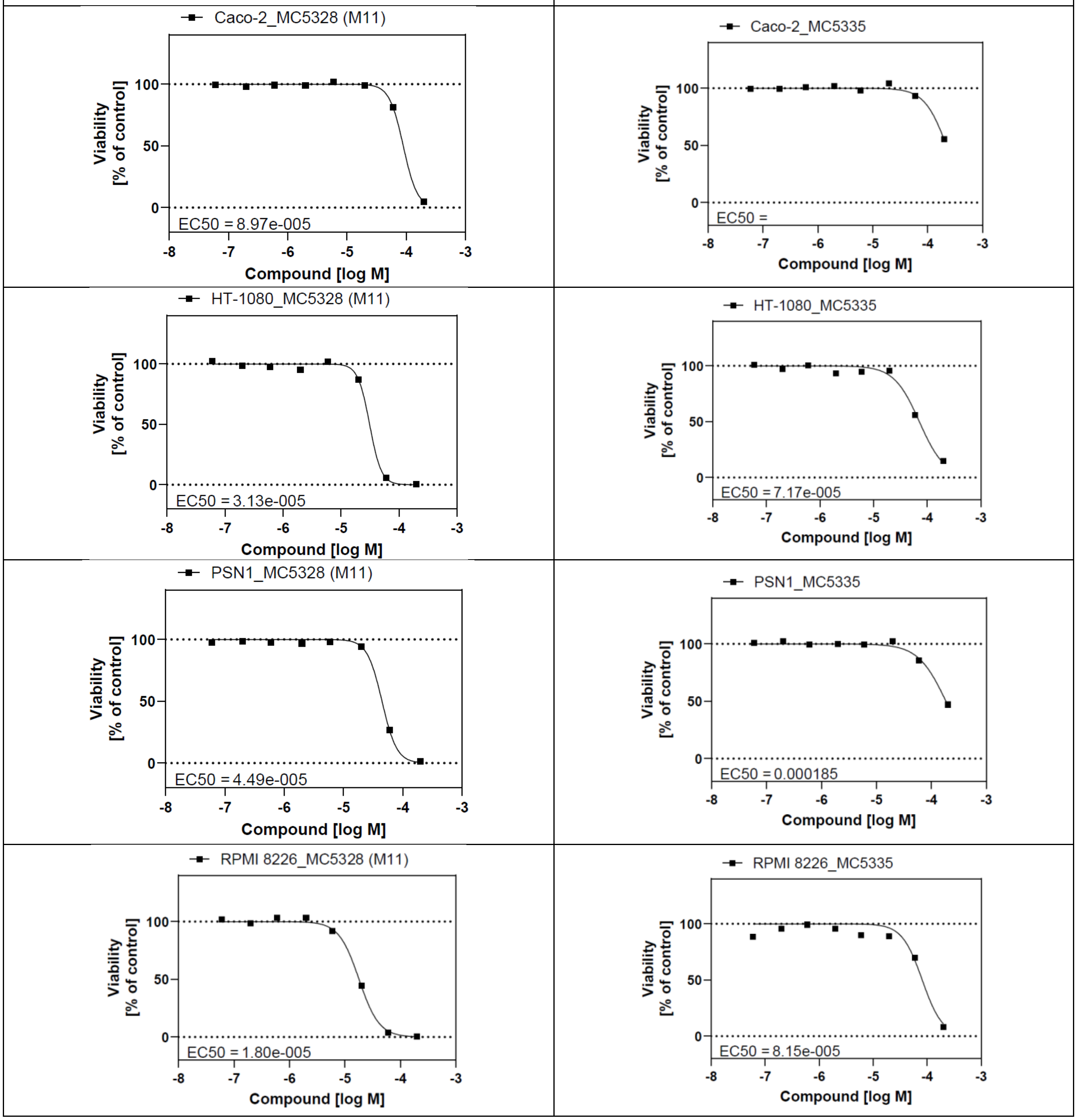

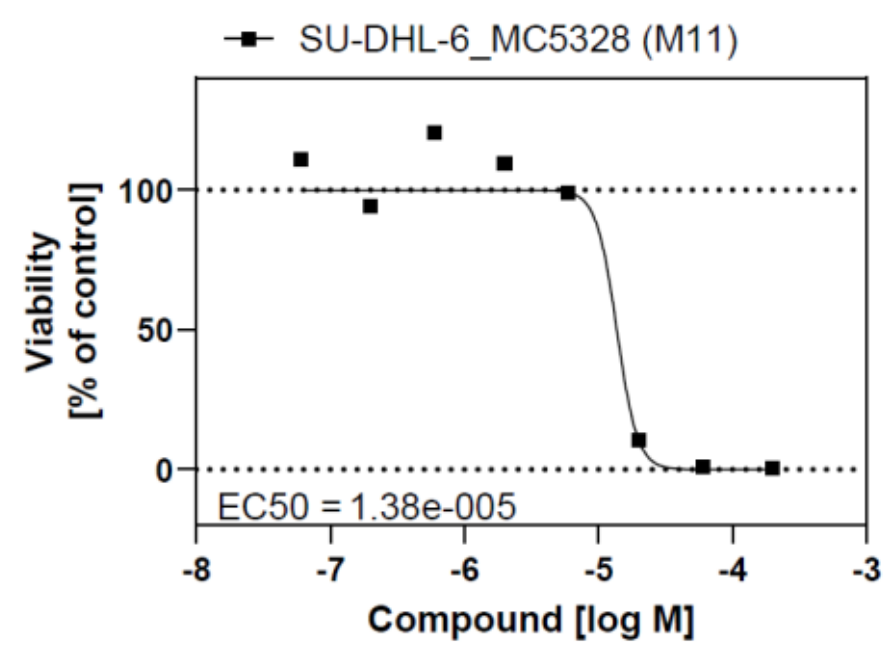

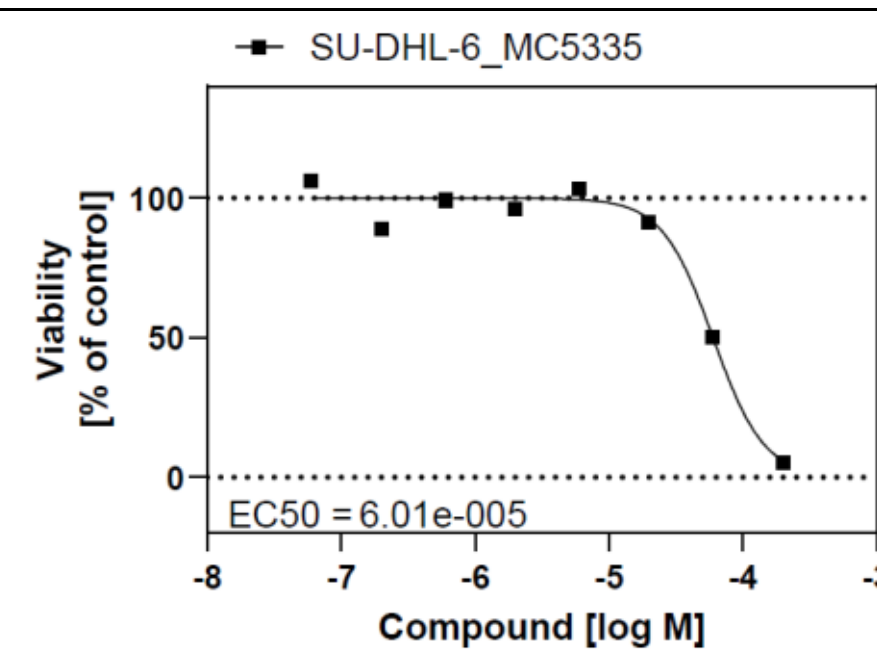

References

(1) Passaro, S.; Corso, G.; Wohlwend, J.; Reveiz, M.; Thaler, S.; Somnath, V. R.; Getz, N.; Portnoi, T.; Roy, J.; Stark, H.; et al. Boltz-2: Towards Accurate and Efficient Binding Affinity Prediction. *bioRxiv* **2025**. DOI: 10.1101/2025.06.14.659707 From NLM PubMed-not-MEDLINE.
(2) Cui, C.; Jiang, M.; Jain, N.; Das, S.; Lo, Y. H.; Kermani, A. A.; Pipatpolkai, T.; Sun, J. Structural basis of human NOX5 activation. *Nat Commun* **2024**, *15* (1), 3994. DOI: 10.1038/s41467-024-48467-y From NLM Medline.
(3) Gazizov, A.; Lian, A.; Goverde, C.; Mou, J.; Ovchinnikov, S.; Polizzi, N. F. AF2BIND: predicting small-molecule binding sites using the pair representation of AlphaFold2. *Nat Methods* **2026**, *23* (3), 626-635. DOI: 10.1038/s41592-026-03011-2 From NLM Medline.
(4) Schake, P.; Bolz, S. N.; Linnemann, K.; Schroeder, M. PLIP 2025: introducing protein-protein interactions to the protein-ligand interaction profiler. *Nucleic Acids Res* **2025**, *53* (W1), W463-W465. DOI: 10.1093/nar/gkaf361 From NLM Medline.
(5) Mosmann, T. Rapid colorimetric assay for cellular growth and survival: application to proliferation and cytotoxicity assays. *J Immunol Methods* **1983**, *65* (1-2), 55-63. DOI: 10.1016/0022-1759(83)90303-4 From NLM Medline.
(6) Lowry, O.; Rosebrough, N.; Farr, A. L.; Randall, R. PROTEIN MEASUREMENT WITH THE FOLIN PHENOL REAGENT. *Journal of Biological Chemistry* **1951**, *193* (1), 265-275. DOI: https://doi.org/10.1016/S0021-9258(19)52451-6.
(7) Taupin, P.; Zini, S.; Cesselin, F.; Ben-Ari, Y.; Roisin, M. P. Subcellular fractionation on Percoll gradient of mossy fiber synaptosomes: morphological and biochemical characterization in control and degranulated rat hippocampus. *J Neurochem* **1994**, *62* (4), 1586-1595. DOI: 10.1046/j.1471-4159.1994.62041586.x From NLM Medline.
(8) Stokes, A. H.; Freeman, W. M.; Mitchell, S. G.; Burnette, T. A.; Hellmann, G. M.; Vrana, K. E. Induction of GADD45 and GADD153 in neuroblastoma cells by dopamine-induced toxicity. *Neurotoxicology* **2002**, *23* (6), 675-684. DOI: 10.1016/S0161-813X(02)00093-1 From NLM Medline.
(9) Mungarro-Menchaca, X.; Ferrera, P.; Moran, J.; Arias, C. beta-Amyloid peptide induces ultrastructural changes in synaptosomes and potentiates mitochondrial dysfunction in the presence of ryanodine. *J Neurosci Res* **2002**, *68* (1), 89-96. DOI: 10.1002/jnr.10193 From NLM Medline.

(10) Robyt, J. F.; Ackerman, R. J.; Chittenden, C. G. Reaction of protein disulfide groups with Ellman's reagent: a case study of the number of sulfhydryl and disulfide groups in Aspergillus oryzae -amylase, papain, and lysozyme. *Arch Biochem Biophys* **1971**, *147* (1), 262-269. DOI: 10.1016/0003-9861(71)90334-1 From NLM Medline.
(11) Ravindranath, V.; Anandatheerthavarada, H. K. Preparation of brain microsomes with cytochrome P450 activity using calcium aggregation method. *Anal Biochem* **1990**, *187* (2), 310-313. DOI: 10.1016/0003-2697(90)90461-h From NLM Medline.
(12) Mansuy, D.; Sassi, A.; Dansette, P. M.; Plat, M. A new potent inhibitor of lipid peroxidation in vitro and in vivo, the hepatoprotective drug anisyldithiolthione. *Biochem Biophys Res Commun* **1986**, *135* (3), 1015-1021. DOI: 10.1016/0006-291x(86)91029-6 From NLM Medline.
(13) Deby, C.; Goutier, R. New perspectives on the biochemistry of superoxide anion and the efficiency of superoxide dismutases. *Biochem Pharmacol* **1990**, *39* (3), 399-405. DOI: 10.1016/0006-2952(90)90043-k From NLM Medline.
(14) Sims, N. R.; Anderson, M. F. Isolation of mitochondria from rat brain using Percoll density gradient centrifugation. *Nat Protoc* **2008**, *3* (7), 1228-1239. DOI: 10.1038/nprot.2008.105 From NLM Medline.
(15) Karlsson, J.; Emgard, M.; Brundin, P.; Burkitt, M. J. trans-resveratrol protects embryonic mesencephalic cells from tert-butyl hydroperoxide: electron paramagnetic resonance spin trapping evidence for a radical scavenging mechanism. *J Neurochem* **2000**, *75* (1), 141-150. DOI: 10.1046/j.1471-4159.2000.0750141.x From NLM Medline.
(16) Shirani, M.; Alizadeh, S.; Mahdavinia, M.; Dehghani, M. A. The ameliorative effect of quercetin on bisphenol A-induced toxicity in mitochondria isolated from rats. *Environ Sci Pollut Res Int* **2019**, *26* (8), 7688-7696. DOI: 10.1007/s11356-018-04119-5 From NLM Medline.
(17) Sadowska-Bartosz, I.; Galiniak, S.; Bartosz, G. Modification of the deoxyribose test to detect strong iron binding. *Acta Biochim Pol* **2017**, *64* (1), 195-198. DOI: 10.18388/abp.2016_1385 From NLM Medline.
(18) Genaro-Mattos, T. C.; Dalvi, L. T.; Oliveira, R. G.; Ginani, J. S.; Hermes-Lima, M. Reevaluation of the 2-deoxyribose assay for determination of free radical formation. *Biochim Biophys Acta* **2009**, *1790* (12), 1636-1642. DOI: 10.1016/j.bbagen.2009.09.003 From NLM Medline.
(19) Roda, A.; Guardigli, M.; Pasini, P.; Mirasoli, M. Bioluminescence and chemiluminescence in drug screening. *Anal Bioanal Chem* **2003**, *377* (5), 826-833. DOI: 10.1007/s00216-003-2096-6 From NLM Medline.
(20) Tzani, M. A.; Gioftsidou, D. K.; Kallitsakis, M. G.; Pliatsios, N. V.; Kalogiouri, N. P.; Angaridis, P. A.; Lykakis, I. N.; Terzidis, M. A. Direct and Indirect Chemiluminescence: Reactions, Mechanisms and Challenges. *Molecules* **2021**, *26* (24). DOI: 10.3390/molecules26247664 From NLM PubMed-not-MEDLINE.
(21) Khan, P.; Idrees, D.; Moxley, M. A.; Corbett, J. A.; Ahmad, F.; von Figura, G.; Sly, W. S.; Waheed, A.; Hassan, M. I. Luminol-based chemiluminescent signals: clinical and non-clinical application and future uses. *Appl Biochem Biotechnol* **2014**, *173* (2), 333-355. DOI: 10.1007/s12010-014-0850-1 From NLM Medline.
(22) Ameziane-El-Hassani, R.; Talbot, M.; de Souza Dos Santos, M. C.; Al Ghuzlan, A.; Hartl, D.; Bidart, J. M.; De Deken, X.; Miot, F.; Diallo, I.; de Vathaire, F.; et al. NADPH oxidase DUOX1 promotes long-term persistence of oxidative stress after an exposure to irradiation. *Proc Natl Acad Sci U S A* **2015**, *112* (16), 5051-5056. DOI: 10.1073/pnas.1420707112 From NLM Medline.
